# Mimicking associative learning using an ion-trapping non-volatile synaptic organic electrochemical transistor

**DOI:** 10.1038/s41467-021-22680-5

**Published:** 2021-04-30

**Authors:** Xudong Ji, Bryan D. Paulsen, Gary K. K. Chik, Ruiheng Wu, Yuyang Yin, Paddy K. L. Chan, Jonathan Rivnay

**Affiliations:** 1 Department of Mechanical Engineering, The University of Hong Kong, Pok Fu Lam, Hong Kong; 2 Department of Biomedical Engineering, Northwestern University, Evanston, IL USA; 3 Simpson Querrey Institute, Northwestern University, Chicago, IL USA; 4 Advanced Biomedical Instrumentation Centre, Hong Kong Science Park, Shatin, New Territories Hong Kong

**Keywords:** Electrical and electronic engineering, Electronic devices

## Abstract

Associative learning, a critical learning principle to improve an individual’s adaptability, has been emulated by few organic electrochemical devices. However, complicated bias schemes, high write voltages, as well as process irreversibility hinder the further development of associative learning circuits. Here, by adopting a poly(3,4-ethylenedioxythiophene):tosylate/Polytetrahydrofuran composite as the active channel, we present a non-volatile organic electrochemical transistor that shows a write bias less than 0.8 V and retention time longer than 200 min without decoupling the write and read operations. By incorporating a pressure sensor and a photoresistor, a neuromorphic circuit is demonstrated with the ability to associate two physical inputs (light and pressure) instead of normally demonstrated electrical inputs in other associative learning circuits. To unravel the non-volatility of this material, ultraviolet-visible-near-infrared spectroscopy, X-ray photoelectron spectroscopy and grazing-incidence wide-angle X-ray scattering are used to characterize the oxidation level variation, compositional change, and the structural modulation of the poly(3,4-ethylenedioxythiophene):tosylate/Polytetrahydrofuran films in various conductance states. The implementation of the associative learning circuit as well as the understanding of the non-volatile material represent critical advances for organic electrochemical devices in neuromorphic applications.

## Abstract (web summary)

Organic transistors that can simulate basic synaptic functions and act as biomimetic devices are advantageous for next generation bioelectronics. Here, the authors realize non-volatile organic electrochemical transistors with optimized performance required for associative learning circuits.

## Introduction

The human brain is a complex computing machine that contains ~10^11^ neurons interconnected by ~10^15^ synapses^1,2^. It works in a highly parallel, fault-tolerant, and energy-efficient manner that can easily outperform modern computers in some complex and unstructured tasks, such as pattern recognition, motor control, and multisensory integration^3–6^. Inspired by the operation of human brain, synaptic devices have attracted widespread interest in recent years^7–10^, and have great potential to serve as building blocks for non-von Neumann neuromorphic computing in the next-generation artificial intelligence. As one of the most well-studied components of synaptic devices, inorganic memristors based on various switching mechanisms have been developed and extensively adopted as artificial synapses^11–16^. However, application challenges including non-linearity in the write process, energy-costly switching, and lack of biocompatibility for tissue interfacing still exist^17,18^, limiting their implementation. Organic synaptic transistors present a natural alternative: they show low voltage writing^19^, continuous tuning of conductance states^20^, materials tunability, and biocompatibility^21^ as well as potential for low-cost and large-area manufacturing^22^. Based on these unique properties, organic synaptic transistors can not only simulate basic synaptic functions like short-term plasticity (STP)^23^, long-term plasticity (LTP)^24^, and spiking time-dependent plasticity (STDP)^25^ but are also able to function as biomimetic devices to directly interface with living tissue^26,27^ which is critical for next-generation bioelectronics capable on high order signal processing and analysis.

Simulating the learning process of the human brain such as associative learning is important in bioelectronics and brain–computer interface (BCI) research. As a fundamental learning principle, associative learning is particularly important for an individual’s adaptability^28^. The best-known associative learning experiment is the Pavlov’s dog experiment, which claims that the conditioned stimulus (CS, ring of bell) can only trigger the unconditioned response (UR, salivation of dog) after the dog has undergone training sessions incorporating both CS and unconditioned stimulus (US, sign of food). To simulate the associative learning, an organic synaptic transistor with non-volatile memory property whose conductance state can be tuned during training is essential. van de Burgt et al. developed an electrochemical neuromorphic organic device (ENODe) whose conductance can be stabilized by decoupling the write and read process, which can be achieved by either physically disconnecting the gate electrode^20^, or by introducing an access device like conductive bridge memory (CBM) at the gate terminal^29^. Their device shows extreme low switching energy <10 pJ. However, in this device, the presynaptic terminal (gate) must be decoupled from the channel in order to maintain non-volatility when simulating associative learning. As a result, additional access devices or high input impedance on gate terminal is necessary, which complicates the circuit design. Gerasimov et al. achieved associative learning by adopting the in situ polymerization of monomer to alter the conductance in the channel of the transistor^30^. However, the monomer contained electrolyte and the irreversible polymerization process limits the potential of this approach. Another approach has been employed whereby a large bias (~5 V) is usually necessary to induce the heavily electrochemical doping in the active channel of the transistor, the slow ion migration kinetics, or even the irreversible electrochemical doping change the conductance of the transistor and enable the associative learning^31–33^. However, large write bias, incomplete reversibility, and limited retention time are drawbacks. Based on the above-mentioned concerns about using organic synaptic transistors to simulate associative learning, a non-volatile transistor with write bias <1 V, coupled write and read operation, reversible conductance tuning and long retention time is highly desired in an associative learning circuit.

Herein, an organic electrochemical transistor (OECT), a mixed ionic/electronic device often employed for applications in bioelectronics^34–36^, has been used as the pivotal component in the associative learning circuit with a previously reported vapor phase polymerized poly(3,4-ethylenedioxythiophene):tosylate (PEDOT:Tos)/ Polytetrahydrofuran (PTHF) composite as the active layer^37,38^. The PEDOT:Tos/PTHF-based OECT with photolithographically patterned channel show much faster response time (~1 ms) compared with previously demonstrated device due to over 2000-fold channel size reduction^38^. At the same time, the OECT shows non-volatile characteristics with write bias <0.8 V, reversible and continuous conductance tuning ability as well as long retention time (>200 min) in a coupled write and read testing scheme. Forms of synaptic plasticity like paired-pulse facilitation (PPF), post-tetanic potentiation (PTP), and short-term memory (STM) to long-term memory (LTM) transition have been simulated and found critically related to the mass ratios between PEDOT:Tos and PTHF. Different from other neuromorphic device-based associative learning circuits where two electrical inputs^20,31,39^ or one electrical input together with one physical input^40,41^ are usually involved, the synaptic circuit described herein can associate two physical inputs (light and pressure) by integrating a pressure sensor and a photoresistor with a volatile and a non-volatile OECT. Finally, the oxidation level, composition, and microstructure of PEDOT:Tos/PTHF composites under electrical bias were intensively evaluated by spectroscopy and scattering to unravel the origin of non-volatility. The non-volatile OECT with superior memory retention and the synaptic circuit with associative learning functions will have a significant impact in the next-generation neuromorphic devices and bioelectronics such as neuroprosthetics, which could help patients with self-perception and learning challenges.

## Results

### Electrical characterization of non-volatile OECT

**Fig. 1:**
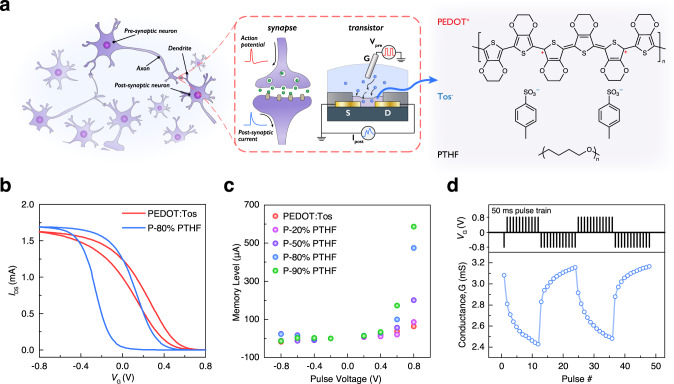
Electrical non-volatility of OECT. **a** Analogy of synapse and OECT and the chemical structure of PEDOT^+^, Tos^−^, and PTHF. **b** Transfer characteristics of the PEDOT:Tos-based OECT and the P-80% PTHF-based OECT ($W=500\,\upmu{\rm{m}},\,L=10\,\upmu{\rm{m}}$). Optical micrograph of channel is shown in Supplementary Fig. 4a. **c** Memory level with respect to channel composition of OECT and programmed gate voltage. **d** Reversible conductance change of P-80% PTHF-based OECT in response to a pulse train with different polarity (conductance value calculated from Supplementary Fig. 8b).

A synapse is a structure through which a neuron can transmit signals to another neuron. The action potential in the presynaptic neuron triggers the release of neurotransmitters from synaptic vesicles into the synaptic cleft. Neurotransmitters will then bind to receptors at the plasma membrane of the postsynaptic neuron and generate the postsynaptic current. Analogously, OECT gate voltage and channel current can be regarded as the action potential and postsynaptic current, respectively. Similarly, the electrochemical doping and dedoping process in an OECT channel by the voltage-controlled injection and extraction of ions is quite similar to the neurotransmitter release and uptake at the synaptic cleft (Fig. 1a). Before simulating synaptic functions using OECT, the non-volatility of OECTs is evaluated first. OECTs were fabricated photolithographically (Supplementary Fig. 1) with PEDOT:Tos/PTHF composites (insert in Fig. 1a), which is deposited by vapor phase polymerization (VPP, Supplementary Fig. 2), as active channel. Different mass ratios of PTHF were adopted and the resulting films are annotated as P-x% PTHF (x = 0, 20, 50, 80, 90) (in the film, x% is PTHF by mass). The morphology and thickness of P-x% PTHF films are shown in Supplementary Fig. 3. The transfer characteristics of the OECTs were measured by cycling *V*_G_ from −0.8 to 0.8 V while maintaining *V*_DS_ at −0.2 V. By comparing the transfer curves of the PEDOT:Tos-based OECT with the P-80% PTHF-based OECT in Fig. 1b, a significant hysteresis enhancement with a memory window around 0.39 V could be observed in the P-80% PTHF-based OECT. Supplementary Fig. 4b confirms that OECTs of higher PTHF blend ratios show larger hysteresis, i.e., memory effect. To validate the cycling stability of the hysteresis, a P-80% PTHF-based OECT was consecutively scanned 50 times between −0.8 V and 0.8 V and showed repeatable characteristics (Supplementary Fig. 5). By carefully controlling the VPP process, the reproducibility of the fabrication is good and the P-80% PTHF-based OECTs show coherent performance with maximum current values of $1.12\pm 0.27\,{\rm{mA}}$ and a memory window of $0.34\pm 0.07\,{\rm{V}}$ (*N* = 6, over 3 separate VPP syntheses). To investigate the non-volatile feature of the PEDOT:Tos/PTHF-based OECTs, the charge retention property was further studied by applying 1 s pulsed gate voltage with various amplitudes and polarities. The memory level indicated in Fig. 1c was defined as the magnitude of the difference in the steady-state channel current after being programmed at a specific gate voltage (${I}_{{\rm{DS}}\_{\rm{Steady}}({\rm{Vg}})}$) relative to the steady-state channel current after being programmed at the initial −0.2 V gate voltage (${I}_{{\rm{DS}}\_{\rm{Steady}}(-0.2)}$) (Supplementary Fig. 6). As shown in Fig. 1c, the memory level increased with increasing positive programmed gate voltage and increasing PTHF blend ratio, indicating the continuous conductance tunability of the non-volatile OECT with write bias <0.8 V. Charge retention time longer than 200 min was also demonstrated in OECTs with varied biasing condition (0.6, 0.7, and 0.8 V) and channel composition (P-50% PTHF and P-80% PTHF) (Supplementary Fig. 7). The reversibility of the device is critical and was verified in P-80% PTHF-based OECT by programming the device with discrete pulsed voltage or a pulse train as shown in Supplementary Fig. 8. The conductance of the device can be reliably tuned to discrete levels between high and low (Fig. 1d), which implies good reversibility. More importantly, our non-volatile OECT retains its memory effect even when the gate terminal remains connected and the bias is returned to 0 V. As such, this device avoids the use of a transistor or memristor-based access device, which can simplify circuit design in more complicated 1-transistor-based active arrays^42^.

### Mimicking STP using an OECT artificial synapse

**Fig. 2:**
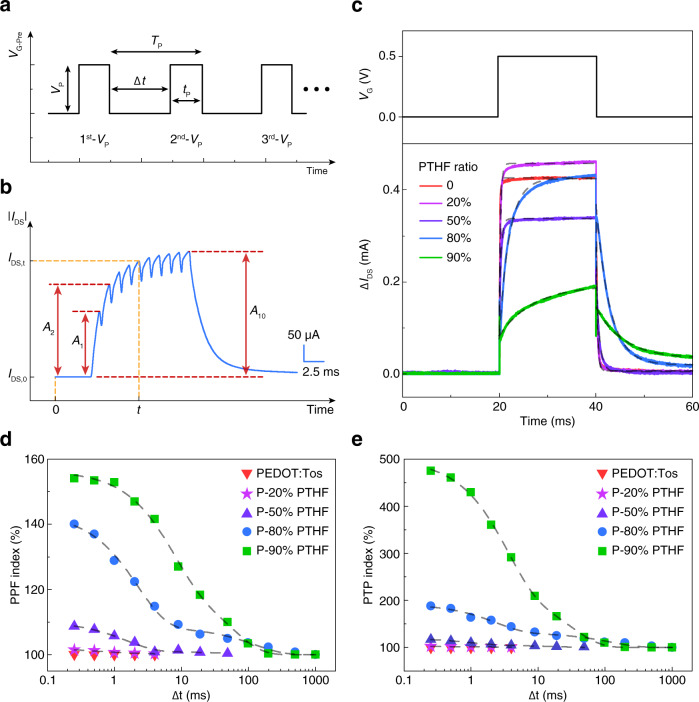
Mimicking STP using an OECT artificial synapse. **a** Patterns of pulsed gate voltage with different parameters; *V*_p_: voltage amplitude, $\triangle t$: pulsed voltage interval time, *t*_p_: pulsed voltage duration, *T*_p_: pulsed voltage period. **b** Channel current modulation induced by 10 consecutive gate voltage pulses in the P-80% PTHF-based OECT. **c** Transient response of the PEDOT:Tos/PTHF-based OECT with different PTHF blend ratio. All the dashed lines are exponential decay fitting curves. The rise-time of the current pulse when the gate voltage was applied is defined as response time, while the decay-time of the current pulse after the gate voltage was removed is defined as ion relaxation time. The response time of devices with different PTHF blend ratio are ${\tau }_{{\rm{res}}\_0}=0.143\,{\rm{ms}},{\tau }_{{\rm{res}}\_20}=0.269\,{\rm{ms}},{\tau }_{{\rm{res}}\_50}=0.387{\rm{ms}},\,{\tau }_{{\rm{res}}\_80}=2.104\,{\rm{ms}},\,{\tau }_{{\rm{res}}\_90}=7.113\,{\rm{ms}}$. The ion relaxation time of devices with different PTHF blend ratio are ${\tau }_{{\rm{rel}}\_0}=0.288\,{\rm{ms}},\,{\tau }_{{\rm{rel}}\_20}=0.241\,{\rm{ms}},{\tau }_{{\rm{rel}}\_50}=0.692{\rm{ms}},\,{\tau }_{{\rm{rel}}\_80}=2.824\,{\rm{ms}},\,{\tau }_{{\rm{rel}}\_90}=5.659\,{\rm{ms}}$. **d**, **e** Paired-pulse facilitation (PPF) and post-tetanic potentiation (PTP) indexes as a function of OECT channel composition and interval time of gate pulses, under *t*_p_ of 1 ms. Gray dashed lines are the fitting curves.

Taking advantage of the true non-volatile behavior of PEDOT:Tos/PTHF composite, artificial synapses based on OECTs were constructed to mimic the synaptic function in the brain. STP, which is critical in information processing and various computational tasks in human brain, was emulated by OECT first. Two notable forms of STP are PPF and PTP, which play key roles in decoding temporal information in auditory or visual signals. To simulate PPF and PTP in non-volatile OECTs, a pulsed voltage on the Ag/AgCl gate electrode was applied with different patterns defined as shown in Fig. 2a. Ten voltage pulses were applied to the gate terminal with an amplitude, *V*_p_, of 0.6 V, a duration (*t*_p_) of 1 ms, and a frequency of 800 Hz. The positive gate voltage de-doped the active channel and decreased the channel current. For simplicity in comparing among different devices, the $\triangle {I}_{{\rm{DS}}}$ was defined here as the absolute value of the channel current variation between the steady-state channel current before applying the gate voltage (${I}_{{\rm{DS}},0}$) and the channel current at a specific time (${I}_{{\rm{DS}},{\rm{t}}}$),

1 $$\triangle {I}_{{\rm{DS}}}=\left|{I}_{{\rm{DS}},0}-{I}_{{\rm{DS}},{\rm{t}}}\right|$$

Fig. 2b shows the triggered $\triangle {I}_{{\rm{DS}}}$ in the P-80% PTHF-based OECT by a pulsed gate voltage. The two performance indicators, PPF and PTP indexes, were defined from Fig. 2b as:

2 $${\rm{PPF}}\,{\rm{index}}=({A}_{2}/{A}_{1}\times 100) \%$$

3 $${\rm{PTP}}\,{\rm{index}}=({A}_{10}/{A}_{1}\times 100) \%$$

The amplitudes of the second current pulse in PPF and the tenth current pulse in PTP were compared to that of the first one. This facilitation effect was stronger when increasing the frequency of the presynaptic voltage pulses or when decreasing their interval time, as illustrated in Supplementary Fig. 9. The PPF and PTP behaviors were highly correlated with the relaxation time of the OECT devices. When the relaxation time was longer than the pulse interval time, the cations in the OECT channel triggered by the first pulse were unable to completely diffuse back to the electrolyte, and these accumulated cations continued to maintain the de-doped state of the channel and tune its current with the addition of cations injected by successive pulses^21^. As a result, the next current pulse was facilitated when the presynaptic voltage pulses had a short interval time. Because the charge relaxation time varied markedly with the different PTHF blend ratios in the OECTs’ active channels (Fig. 2c), the PPF and PTP behaviors were strongly dependent on the PTHF ratio. The relationship between the PPF and PTP indexes with the presynaptic pulse voltage interval time, and the OECT channel composition are shown in Fig. 2d and e. Both the PPF and PTP showed a typical two-phase exponential behavior and can be fitted by a double-exponential function:

4 $${\rm{PPF}}\,{\rm{index}},{\rm{PTP}}\,{\rm{index}}=1+{C}_{1}\,\exp \,\left(\frac{-t}{{\tau }_{1}}\right)+{C}_{2}\,\exp \,\left(\frac{-t}{{\tau }_{2}}\right)$$

where $t$ is the pulse interval time, ${C}_{1}$ and ${C}_{2}$ were the initial facilitation magnitudes of each phase, and ${\tau }_{1}$ and ${\tau }_{2}$ were the respective relaxation time constant. Interestingly this two-phase exponential decay is coherent with the decay of synaptic facilitation in a biological synapse; ${\tau }_{1}$ and ${\tau }_{2}$ can be used to mimic the relaxation time of rapid and slower decay phase in a synapse^43^. However, the fundamental physical meaning of these two time constants in this particular system requires further investigation. From fitting parameters listed in Supplementary Tables 1 and 2, one can notice that the facilitation magnitude (${C}_{1}$ and ${C}_{2}$) and relaxation time (${\tau }_{1}$ and ${\tau }_{2}$) generally follow the trend that the elevated value can be obtained with the increment of the PTHF blend ratio. However, one exception is the relaxation time ${\tau }_{2}$ in P-90 PTHF sample. This phenomenon was attributed to the slow response time of P-90% PTHF which will be discussed further below.

### Mimicking LTP using an OECT artificial synapse

**Fig. 3:**
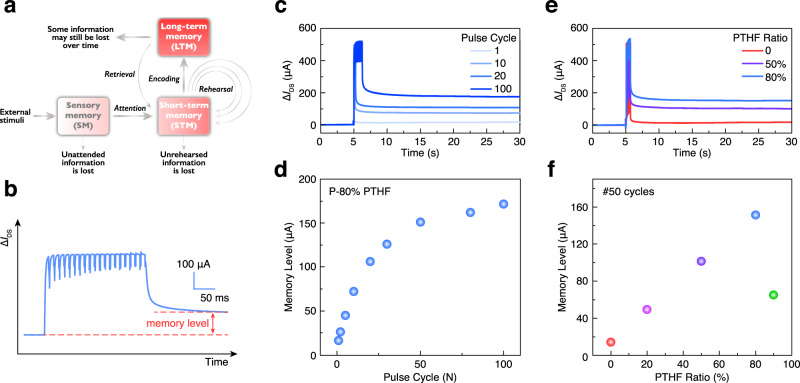
Mimicking LTP using an OECT artificial synapse. **a** Scheme of the memory formation process in the human brain. **b** Short-term memory (STM) to long-term memory (LTM) transition in the P-80% PTHF-based OECT triggered by 20 consecutive gate voltage pulses. **c** Channel current change comparison of the P-80% PTHF-based OECT after biasing by four different cycles of gate voltage pulses. **d** Memory level as a function of pulsed gate voltage training cycles. **e** Channel current change comparison of OECT among three different PTHF ratio after biasing by the 50 pulsed gate voltage. **f** Memory level with respect to channel composition of OECT at 50 pulsed gate voltage.

In contrast to STP, in which temporary modifications of synaptic weight occur, LTP plays its role in memory or learning by changing the synaptic weight more persistently. LTP can last longer from minutes to days or even years. The process of memory formation in the brain (from sensory memory (SM) to STM and finally to LTM) can be aided by repeated rehearsal or training and is illustrated in Fig. 3a. The transition process from STM to LTM was simulated in our non-volatile OECTs with the repeated application of presynaptic gate voltage pulses, which was analogous to the rehearsal process, and the LTM level in OECTs was defined as shown in Fig. 3b. The dedoping level of the channel material during 20 pulsing cycles is believed to be increased after plotting *I*_DS_ on a log-scale and considering the magnitude of the leakage current (*I*_G_), which begins to dominate at the applied potentials of interest. This effect will influence the precise evaluation of electronic current inside the channel (indicative of the dedoping level) (Supplementary Fig. 10). A series of pulsed gate voltages with fixed pulse amplitude (0.7 V), duration (10 ms), and interval time (2.5 ms) were applied to the P-80% PTHF-based OECT. The changes in channel current ($\triangle {I}_{{\rm{DS}}}$) of the OECT device after four representative training cycles are shown in Fig. 3c. As observed in Fig. 3d, the memory level, defined as the steady-state channel current change before and after application of gate voltage, increased with each additional training cycle of pulsed gate voltage, which indicated a transition from STM to LTM. This phenomenon is very similar to the process that occurs in a neural synapse, in which a large number of neurotransmitters can be released when the presynaptic neuron is triggered by several action potentials, enhancing the transmission efficiency between neurons. Besides the effect of training cycles on the LTM, we also study the influence of channel composition on the LTM. A comparison among the PEDOT:Tos, the P-50% PTHF and the P-80% PTHF-based OECT after training with 50 consecutive pulses (Fig. 3e) shows a clear STM to LTM transition for the P-50% and P-80% PTHF devices, while in the device without PTHF, almost all STM was lost and was not transferred to LTM after removing the pulsed gate voltage. The slightly observable memory effect in PEDOT:Tos-based OECTs may be due to a small population of residual ions or to a bias-stress effect during testing. The relation between memory level and channel composition at 50 training cycles is shown in Fig. 3f. As opposed to the monotonic increase of memory level vs composition we observed in Fig. 1c, memory level here increased non-monotonically with larger PTHF fractions. When the blend ratio of PTHF increased from 0 to 80%, the memory level increased with the PTHF blend ratio. Beyond P-80%, the memory level decreased. Based on this observation and PPF and PTP results obtained for the P-90% devices (the relaxation time ${\tau }_{2}$ in the P-90% PTHF-based OECT is smaller than the one in the P-80% PTHF-based OECT), we deduce that an excessively high PTHF blend ratio (90%) results in a thicker film (~1.6 μm, Supplementary Fig. 3j) and thus slower response time (~7 ms, Fig. 2c). Given the short pulse duration (10 ms) in STM to LTM measurement, this gives the illusion of lower memory levels, which can be recovered with longer input pulse duration. Thus, balancing the observed memory effect with the response time at this transistor size scale, P-80% PTHF is selected as the optimal material for non-volatile OECTs in the subsequent synaptic circuits.

### OECT-based neuromorphic circuit for demonstrating associative learning

**Fig. 4:**
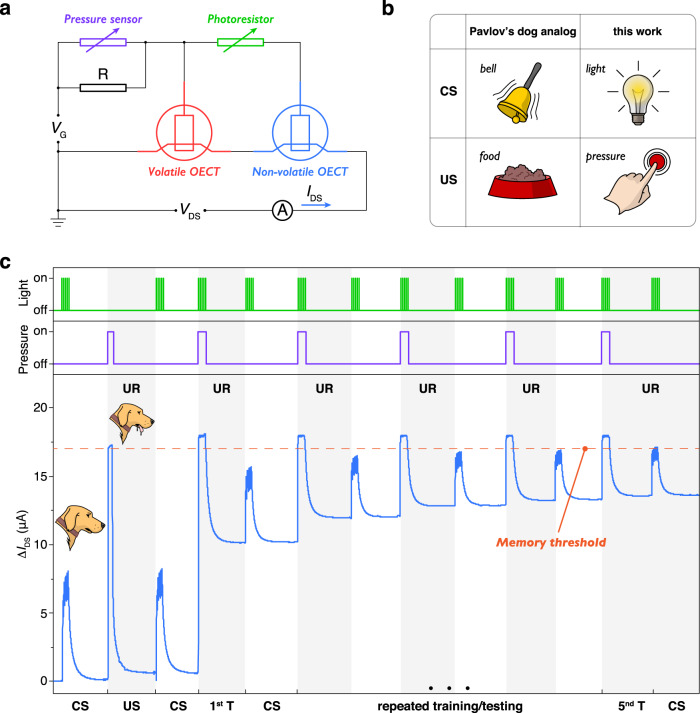
OECT-based neuromorphic circuit for demonstrating associative learning. **a** Circuit diagram of the neuromorphic circuit for simulating associative learning. *V*_G_ = 0.7 V and *V*_DS_ = −0.2 V. **b** Analogy of the CS (conditioned stimulus) and US (unconditioned stimulus) in Pavlov’s dog experiment and our neuromorphic circuit. **c** Complete associative learning process. After five times training, the CS can trigger UR (unconditioned response, salivation of dog).

Based on the demonstrated synaptic behavior of PEDOT:Tos/PTHF-based OECT, we advanced the single synaptic transistor into a synaptic circuit to simulate associative learning. A pressure sensor, a photoresistor, a PEDOT:PSS-based volatile OECT, and a P-80% PTHF-based non-volatile OECT were integrated into a synaptic circuit (Fig. 4a). Pressure and light were sensed by the corresponding sensors and were transferred to the channel conductance change of the non-volatile OECT as shown in Supplementary Figs. 11 and 12, which allowed the simulation of associative learning between two physical inputs. Light from a LED bulb and pressure from a finger press were considered as CS and US, respectively (Fig. 4b), while the current change in the drain-source circuit ($\triangle {I}_{{\rm{DS}}}$) was considered as UR. The LED bulb, the photoresistor, and the pressure sensor were assembled into an associative learning circuit dock as shown in Supplementary Fig. 13a. The complete associative learning process is shown in Fig. 4c. First, pulsed light (CS, Supplementary Fig. 13b) was applied on the circuit; in this case, only a small part of the gate voltage could be applied on both the volatile and non-volatile OECT as the $13\,{\rm{M}}\Omega$ resistor was connected in series to their gate inputs (Fig. 4a). As a result, only a slight increase of $\triangle {I}_{{\rm{DS}}}$ was achieved without reaching memory threshold. Then, the pressure sensor was pressed by the finger as the US signal (Supplementary Fig. 13c). Due to the low resistance of the pressure sensor while pressing with a finger and the high resistance of the photoresistor in a dark environment, most of the gate voltage was applied on the volatile device and very little was applied on the non-volatile one. Therefore, although a high $\triangle {I}_{{\rm{DS}}}$ was attained to trigger the UR (salivating dog), it later decayed leaving no memory effect because of the untriggered non-volatile OECT. After solely applying the US on our circuit, the CS was implemented again and the amplitude of $\triangle {I}_{{\rm{DS}}}$ was comparable with the first time it was triggered by the CS, which was too small to induce UR. This result confirmed that training only with the US did not help our circuit to associate the CS with the US. Next, the CS and US were applied together as the training process (Supplementary Fig. 13d); in this case, the gate voltage could be ideally applied on both the volatile and non-volatile OECT. Consequently, a high enough $\triangle {I}_{{\rm{DS}}}$ to trigger UR was achieved and it was retained due to the memory property of the non-volatile OECT (Supplementary Fig. 14a). This memory effect (${\triangle I}_{{\rm{DS}}1}$) helped the $\triangle {I}_{{\rm{DS}}}$ approach the memory threshold when triggered by the CS only after the first training. By performing 5 training cycles under the simultaneous application of CS and US, the obtained memory level increased significantly (${\triangle I}_{{\rm{DS}}5}$, Supplementary Fig. 14b). Finally, the CS alone was able to trigger UR (Fig. 4c, Supplementary Fig. 14b) without the existence of the US, which confirmed the neuromorphic circuit successfully associated the CS with the US and enabled the learning process in the neuromorphic circuit. The demonstration of associative learning is the first important step, our proposed circuit can be further extended to more sensory inputs (available from a suit of possible sensors) on the non-volatile OECTs and can be potentially integrated with electronics skin, smart robotics, or even in implantable devices to directly interface with neurons and synapses, which has great impact in neuroprosthetics and brain-machine interface.

### Spectroelectrochemistry of the P-x% PTHF films

**Fig. 5:**
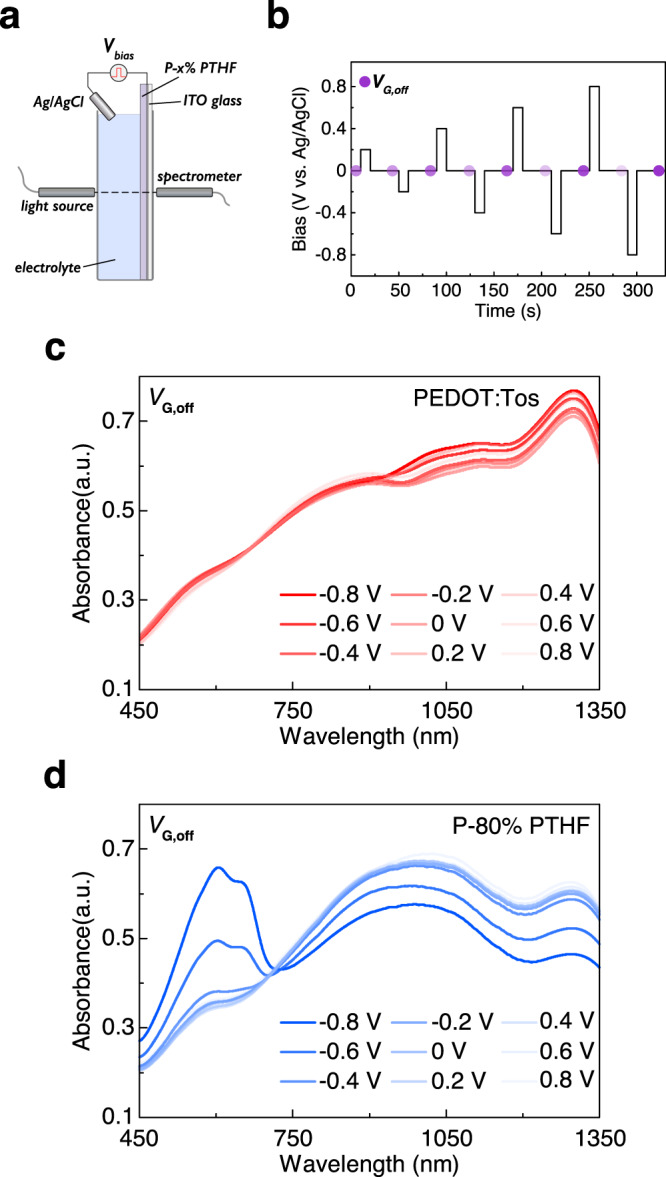
Spectroelectrochemistry of the P-x% PTHF films. **a** Set up of operando spectroelectrochemistry study of PEDOT:Tos/PTHF film with different compositions. **b** The pattern of the stepwise bias that was applied on the working electrode (P-x% PTHF). **c**, **d** UV-Vis-NIR absorbance of PEDOT:Tos and P-80% PTHF after the electrical bias has been removed.

Beyond demonstrating the synaptic functions of the non-volatile OECT, it is key to understand the origin of the non-volatility. Although previous research proposed the structural collapse of PTHF result in the non-volatility of vapor phase polymerized PEDOT with PTHF^37,38^, further characterizations are needed to investigate how the film properties change optically, compositionally, and structurally under various gate bias. Spectroelectrochemistry of the P-x% PTHF films under different biases was investigated by operando ultraviolet-visible-near-infrared (UV-Vis-NIR) spectroscopy as shown in Fig. 5a. P-x% PTHF films were deposited on ITO glass and functioned as the working electrode in an electrochemical cell. A stepwise voltage with 10 s duration was applied between the P-x% PTHF films and an Ag/AgCl pellet (reference/counter electrode) through 0.1 M NaCl electrolyte (Fig. 5b) while the UV-Vis-NIR absorbance was continuously monitored. The UV-Vis-NIR absorbance during electrical bias of PEDOT:Tos and P-80% PTHF are shown in Supplementary Fig. 15. As expected, two samples with a different blend ratio of PTHF showed decreased π–π* transition absorbance (~620 nm) and increased polaronic state absorbance, including polaron (~900 nm) and bipolaron (~1350 nm), which is consistent with electrical properties of two samples being converted from an insulating state (low oxidation level) to a highly conductive state (high oxidation level) when the bias increase from −0.8 V (dedoping) to 0.8 V (doping). However, when comparing the UV-Vis-NIR absorbance after the bias has been returned to 0 V, the samples (Fig. 5c and d) displayed different behavior. Pure PEDOT:Tos film did not retain the spectral signature of the various oxidation levels, while for P-80% PTHF, the distinct residual spectral signatures of the oxidation levels were maintained without bias. When comparing the state with the lowest oxidation level (−0.8 V, 10 s) to the state with the highest oxidation level (0.8 V, 10 s), the modulation of π–π* transition, polaron, and bipolaron absorbance of PEDOT:Tos were all <5%. Conversely, the π–π* transition absorbance of P-80% PTHF increased by 75.9%, the polaron absorbance of P-80% PTHF decrease by 14.1% and bipolaron absorbance of P-80% PTHF decrease by 25.7%, respectively, as shown in Supplementary Fig. 16. This result indicates that the film with 80% PTHF shows strong non-volatility, in agreement with the electrical behavior of the devices above.

To confirm the reversibility of the oxidation level change caused by the electrical bias in P-80% PTHF, a cyclic doping (0.8 V) and dedoping (−0.8 V) bias was applied and the absorbance of P-80% PTHF was recorded after bias. As shown in Supplementary Fig. 17, the highly repeatable absorbance of P-80% PTHF in both low oxidation level (−0.8 V) and high oxidation level (0.8) confirmed the high reversibility during electrical bias of different polarity. Besides, long-term charge retention of P-80% PTHF was also examined optically as a supplement of the above-mentioned charge retention results that has been electrically demonstrated. Through tracking the intensity of π–π* transition with respect to time, a quick erase of memory state right after the removal of bias can be observed (Supplementary Fig. 18a–c). However, after charge stabilization, distinct difference in low oxidation level (−0.8 V) and high oxidation level (0.8 V) can still be observed after the bias was removed for 1 h, which confirms the charge retention time can be longer than 1 h when the film is still immersed in the electrolyte with gate terminal connected (Supplementary Fig. 18d). The state retention time of the P-80% PTHF film was as long as 8 days after being removed from electrolyte and stored in ambient (Supplementary Fig. 19).

The persistence of a stable low oxidation level in P-80% PTHF films implies the inability of positive electronic charge carriers to return to the PEDOT chains. This could arise electronically, that despite the thermodynamic preference of the PEDOT to return to the doped state, electronic charge transport may be so frustrated that positive charge carriers could not reach the PEDOT chains on experimentally relevant timescales. Alternatively, the non-volatility could arise ionically due to the cations being trapped near the PEDOT chains in the film and anions being impeded from reaching those PEDOT chains, thus preventing the dopant stabilization of electronic charge on the PEDOT chains. Either of these processes, or a combination of both, would maintain the de-doped state, resulting in an enhanced π–π* transition absorbance, diminished polaron and bipolaron absorbance, and diminished film conductivity. Frustrated electronic charge transport alone could not account for the non-volatile nature of the low oxidation state, as the conductivity, though low, is non-zero as shown in Supplementary Fig. 6. Considering the capacity of the PEDOT:Tos/PTHF film and film conductivity in low oxidation state, if electronic charge transport was the limiting step, the film would re-dope on much shorter timescales. A far more likely source of non-volatility is frustrated ionic transport, which results in a large number of trapped cations inside the film driven by electrical bias.

### XPS of the P-x% PTHF films

**Fig. 6:**
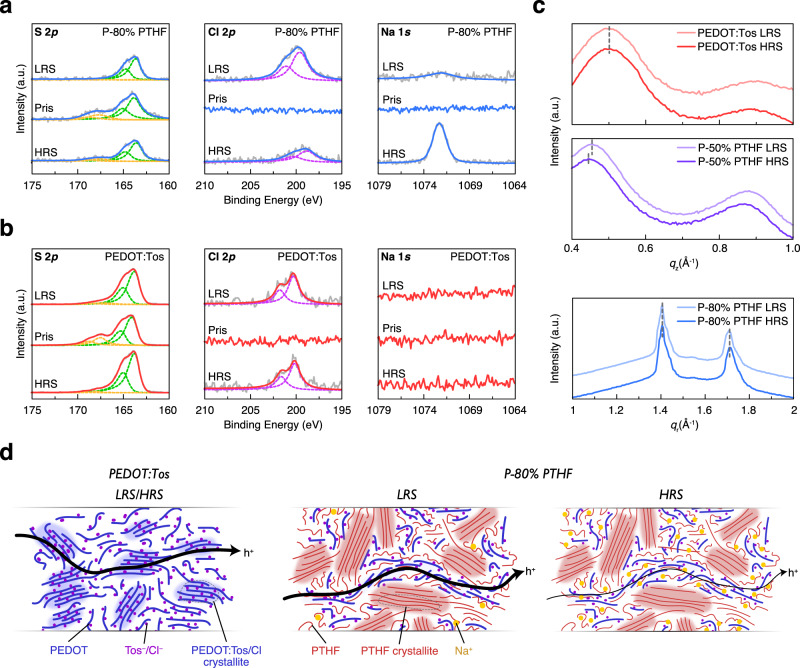
XPS and GIWAXS of the P-x% PTHF films. **a** XPS of the S 2*p*, Cl 2*p*, and Na 1*s* peaks of P-80% PTHF under different conductance states. **b** XPS of the S 2*p*, Cl 2*p*, and Na 1*s* peaks of PEDOT:Tos under different conductance states. In XPS data, gray lines are the original data before fitting. Blue (For P-80% PTHF) and red (for PEDOT:Tos) lines are overall fitted data. Dashed green and yellow lines are fitted doublet peaks of S 2*p* from PEDOT^+^ and Tos^−^, respectively. Dashed purple lines are fitted doublet peaks of Cl 2*p*. **c** Out-of-plane linecuts for PEDOT:Tos and P-50% PTHF in LRS and HRS; and in-plane linecuts for P-80% PTHF in LRS and HRS. **d** Cartoon of the microstructure and composition of PEDOT:Tos and P-80% PTHF in LRS and HRS illustrating the mechanism of non-volatility. PEDOT:Tos has similar microstructure and composition in LRS and HRS, which results in comparable electrical current. For P-80% PTHF, trapped Na^+^ in non-crystalline PTHF fraction leads to a diminished hole current in HRS compared with LRS.

To track the ions inside the P-x% PTHF under different conductance states, X-ray photoelectron spectroscopy (XPS) was used to identify the relative quantity of Tos^−^, Cl^−^, and Na^+^ in the films. The low resistance state (LRS) and high resistance state (HRS) of the films were achieved with 1 min bias of −0.8 and 0.8 V, respectively. As shown in Fig. 6a, pristine P-80% PTHF shows distinct S 2*p* peak from Tos^−^ (166~170 eV) while no Cl 2*p* and Na 1*s* peaks, which is consistent with the composition of pristine P-80% PTHF that the Tos^−^ is the only counterion to dope the PEDOT chain. However, for P-80% PTHF in both LRS and HRS, the S 2*p* peaks from Tos^−^ are diminished while the Cl 2*p* peaks are observed, which is consistent with ion exchange of the more bulky Tos^−^ with the smaller Cl^−^ during electrochemical operation^44^. The same phenomenon is also shown in PEDOT:Tos (Fig. 6b). More importantly, a very strong Na 1*s* peak is observed in P-80% PTHF in HRS compared with the minor Na 1*s* peak in P-80% PTHF in LRS. From the relatively peak area of Na 1*s* and Cl 2*p* in P-80% PTHF in both LRS and HRS, we can conclude that a large amount of Na^+^ is trapped in P-80% PTHF and a part of Cl^−^ is removed during biasing process. At the same time, no Na 1*s* peak is observed in PEDOT:Tos in HRS (Fig. 6b), which confirms that PTHF is indeed the component responsible for Na^+^ trapping. The relative ratio of Na^+^ over Cl^−^ in the bulk area was also calculated from XPS depth profiling (Supplementary Fig. 20); higher Na^+^/Cl^−^ ratio (~0.3) in HRS compared with (<0.1) in LRS confirms that the trapping of Na^+^ occurs in the bulk of the P-80% PTHF film, and not only near the polymer/electrolyte interface.

### GIWAXS of the P-x% PTHF films

GIWAXS was further used to understand the mechanism of non-volatility of P-x% PTHF induced by the trapped Na^+^ from structural point of view. Before conducting GIWAXS on films in different conductance states, understanding the microstructure of the at-cast P-x% PTHF is important (Supplementary Fig. 21). In general, pristine PEDOT:Tos shows a preferred edge-on structure with a lamellar stacking distance 13.7 Å and π–π stacking distance 3.5 Å. The relatively large breadth of the PEDOT:Tos scattering peaks indicate relatively short coherence lengths (<10 nm) of polymer crystallites, which are likely embedded in an amorphous matrix (Supplementary Fig. 22a). Introducing 50% PTHF revealed no new scattering peaks due to PTHF crystallites. Further, PEDOT:Tos scattering peaks showed no appreciable broadening, indicating that the length scales of coherent PEDOT:Tos ordering were not diminished due to the presence of PTHF. The presence of PTHF did bring about shifts in the position of the PEDOT:Tos scattering indicating changes in the PEDOT:Tos d-spacing. The out-of-plane (100) peak shifted to lower *q*_z_, as shown in Supplementary Fig. 23, indicating an increased lamellar stacking distance around 14.4 Å for P-50% PTHF, while the in-plane π-stack peak position remained unchanged. The lamellar expansion may result from PTHF chains being closely integrated with PEDOT chains and partially occupying the inter-lamella space (Supplementary Fig. 22b). However, this lamellar expansion does not account for the total PTHF content added to the composite, suggesting most of the PTHF chains must reside outside the PEDOT crystallites in the amorphous regions along with the amorphous fraction of PEDOT:Tos. When PTHF content was further increased to 80%, PEDOT crystallite scattering peaks could no longer be observed, indicating extensive disruption of PEDOT ordering, or diminished relative scattered intensity that is not resolvable due to isotropic PTHF scattering ((110)*, (020)*)^45,46^. With an 80% PTHF loading, PTHF diffraction shows no shifts in peak position compared to pure PTHF. This result indicates that PEDOT does not disrupt semicrystalline PTHF packing (in P-80% PTHF) and is confined to the amorphous regions of the film (Supplementary Fig. 22c and d).

Based on the results from as-cast P-x% PTHF, we then conduct GIWAXS on P-x% PTHF films in LRS and HRS which are achieved by 1 s bias of −0.8 and 0.8 V, respectively. Because PEDOT:Tos and P-50% PTHF show predominant PEDOT scattering while P-80% PTHF shows intense PTHF scattering, these three compositions are informative for studying the ion-trapping effect both in ordered PEDOT region and PTHF region. The 2D-GIWAXS patterns of PEDOT:Tos, P-50% PTHF, and P-80% PTHF in LRS and HRS are shown in Supplementary Fig. 24. Consistent with the lack of non-volatile state stability, PEDOT:Tos displayed no persisting differences in lattice d-spacing (Fig. 6c), indicating that the rapid electronic relaxation includes a concomitant structural relaxation as illustrated in Supplementary Fig. 25a. However, the P-50% PTHF showed a marked shift in (100) and (200) peak positions (Fig. 6c), consistent with an increase of 0.2 Å in the lamellar spacing in the HRS over the LRS as schematically shown in Supplementary Fig. 25b, which is proposed to be induced by trapped Na^+^. The π-stack associated d-spacing was unchanged for both PEDOT:Tos and P-50% PTHF (Supplementary Fig. 26). Consistent with HRS P-50% PTHF, operando PEDOT:PSS measurements have also shown a lamellar expansion upon dedoping^47^, which indicates electrical stability of the HRS in non-volatile P-50% PTHF should be coupled with a long-lived structural modification. In addition, (020)* and (110)* peaks of PTHF in P-80% PTHF are unchanged in both LRS and HRS (Fig. 6c) which indicates that the PTHF lattice parameters are insensitive to different conductance states. Since switching states necessitates significant ion transport, the lack of change to the PTHF crystallite structure strongly suggests that cations transport and reside within the disordered or aggregated PEDOT:Tos/PTHF fraction. At the same time, the P-80% PTHF has already been demonstrated with even stronger memory effect than P-50% PTHF, implying the crystalline PTHF imposes a further barrier to ionic transport compounding the trapping of Na^+^ in the non-crystalline fraction of PTHF (Fig. 6d). The relevance of the P-50% PTHF samples is thus to highlight the importance of the PTHF chains that are closely interfaced with PEDOT, which presumably exist within P-80% PTHF in a manner not readily resolvable with GIWAXS.

## Discussion

The hindered ionic transport that effectively (reversibly) traps Na^+^ in PTHF may be due to the following reasons: In general, the relatively low oxygen density in the backbone of PTHF (O:C=1:4) reduces the number and proximity of coordination sites for Na^+^ compared with the analog polymer electrolyte polyethylene glycol (PEG) (O:C=1:2). The diminished cation-polymer coordination results in poorer ion solubility and slower ionic transport in the solvent-free condition^48–50^. This is usually overcome in PTHF-based polymer electrolytes with additional solvent like ethylene carbonate (EC)/diethyl carbonate (DEC) that can swell PTHF^50^. The loosely coordinated cation and polymer chain in PTHF will facilitate cation-solvent coordination which enables a faster ionic transport due to the vehicular motion of solvated cation by solvent. However, this phenomenon is not applicable in our device. PTHF is relatively hydrophobic^51^, thus the aqueous electrolyte employed to gate PEDOT:Tos/PTHF OECTs likely imparts minimal swelling or plasticization of the PTHF phase which could facilitate facile ionic transport. Due to the diminished ion-solvent coordination, ion hopping in the backbone of PTHF will dominate. Furthermore, the ion hopping efficiency is reduced due to the low density of ion coordination sites and large distance between them in the PTHF backbone. In addition, the linear PTHF we used also favors crystallization which is determinantal to ionic transport because of the hindered segmental motion of polymer chains^52^. The interpretation here and the above-mentioned characterization provide evidence that the frustrated ionic transport in the non-crystalline PTHF fraction, compounded with increased PTHF crystallinity, cause the trapping of Na^+^ ions, which leads to the observed non-volatility. This mechanism thus provides a materials route to explore new composites for longer state retention, faster response, or selective memory owing to certain ionic species.

In summary, a non-volatile OECT device was successfully fabricated with a VPP deposited PEDOT:Tos/PTHF composite active channel. This device shows fast response time (~1 ms), stable memory window, write bias <0.8 V, reversible and continuous conductance tuning ability as well as long retention time (>200 min) in coupled write and read testing scheme. By varying the pattern of the pulsed gate voltage used as presynaptic input, different synaptic functions from STP, like PPF and PTP, to LTP, like STM to LTM transition, have been emulated. The PTHF ratio in the composite is shown to play a critical role in defining the STP and LTP properties of the device. For STP, a higher blend ratio of PTHF is preferred as reflected by the increased PPF and PTP indexes with elevated PTHF ratios. While for LTP, the memory behavior is not monotonically enlarged with the increased PTHF ratio; the device with 80% PTHF showed the best-attained memory compared with other devices after equivalent training. Based on the non-volatile nature of the OECT, a neuromorphic circuit has been successfully demonstrated for simulating associative learning from two physical inputs by combining a pressure sensor, a photoresistor, a volatile OECT, and a non-volatile OECT. Besides the application of this device, in-depth study of device physic was conducted. A multimodal ex-situ and operando characterization suite is used to investigate the oxidation level variation, compositional change, and the structural modulation of P-x% PTHF films in various biasing conditions. Non-crystalline PTHF is believed to be capable of trapping cations which is the origin of the memory behavior of OECT devices, with PTHF crystallites enhancing the effect. The understanding of the non-volatile material and the application of the associative learning circuit are significant for both fundamental material selection as well as hardware implementation in the area of neuromorphic computing and bioelectronics.

## Methods

### Fabrication of non-volatile OECT device

The device was fabricated by a standard microfabrication technique. 50 nm Au and 5 nm Cr were deposited on a photoresist patterned glass slide by thermal evaporation and the following immersion in DMSO was used to remove the photoresist for defining drain-source pattern of OECT. Two consecutive parylene layers with the same thickness of 1.5 $\upmu{\rm{m}}$ were deposited to encapsulate the metal contact as well as pattern the active layer. The substrate was first treated with 0.5% saline 174 solution to improve the adhesion with the first parylene layer. Before the deposition of the second parylene layer, 2% micro 90 solution was spin-coated as an anti-adhesive layer which enables the delamination of the second parylene layer. Positive photoresist AZ 9260 was patterned on parylene and used as a template to pattern the underneath double-layer parylene by oxygen plasma etching. Before active layer deposition, the photoresist AZ 9260 was stripped by the AZ 400T solution. The device with metal electrodes and patterned parylene layers was transferred to N_2_ filled glove box to avoid moisture-inducing crystallization of oxidant for later polymerization. A mixture solution of oxidant Fe(III):Tos and PTHF in butanol with predefined ratio was spin-coated on device surface followed by 1-min 70 °C thermal annealing. The device was immediately transferred to an EDOT filled tube furnace and the vapor phase polymerization (VPP) of EDOT took place at 70 °C for 1 h with continuous purged N_2_ (100 SCCM). Finally, after the VPP process, the sacrificial parylene layer was peeled off to define the active channel of PEDOT:Tos/PTHF composite. The device was rinsed in DI water for 1 h to remove excess oxidant before doing the electrical measurement.

### Preparation of PEDOT:Tos/PTHF composite precursor

P-80% PTHF is used as an example here. The amount of PTHF was determined by the fact that it needs 2.25 moles of Fe (III) to produce 1 mole PEDOT^53^. 0.37 g PTHF and 1 g Fe(III)Tos were added into 3.63 g butanol to maintain a 20 wt% of Fe(III)Tos in the mixed solution. The mixture was magnetically stirred and heated at 60 °C for 30 min to thoroughly dissolve the PTHF and Fe(III)Tos. Afterward, 60 μL pyridine was added into the mixture as a base inhibitor in the VPP process^54^. A Polytetrafluoroethylene (PTFE)-based filter with pore size 0.4 μm was used to filter the precursor solution before VPP. For PEDOT:Tos/PTHF with other compositions, the detailed mass and volume of each component are listed in Supplementary Table 3 and the mass percentage of Fe(III)Tos is maintained at 20 wt% in all compositions.

### Fabrication of pressure sensor

The detailed fabrication process can be found in our previous publication^55^. In short, the sandpaper was treated with oxygen plasma first and then coated with a self-assembled monolayer (SAM) of Trichloro (1H, 1H, 2H, 2H-perfluorooctyl) saline by vapor phase deposition. This additional hydrophobic SAM can help the demolding of Polydimethylsiloxane (PDMS) later. The mixture of SYLGARD 184 and curing agent (10:1) was spin-coated on sandpaper at 700 rpm and followed by 30-min thermal annealing. After that, the PDMS thin film with micro-hump structure was peeled off and treated with oxygen plasma for 10 min. PEDOT:PSS (CLEVIOS PH 1000) was spin-coated on a PDMS surface at 700 rpm and then dried at 30 °C for 30 min. The PDMS coated with PEDOT:PSS was placed on screen-printed interdigitated silver electrodes to form a pressure sensor. To make sure the pressure is evenly distributed on the pressure sensor, a square of Si wafer was mounted on the back of the PDMS.

### Operando UV-Vis-NIR spectroscopy

Spectroelectrochemistry of P-x% PTHF coated on ITO glass electrode was carried out in 0.1 M aqueous NaCl in a PMMA cuvette with an Ag/AgCl pellet (Warner Instruments) reference/counter electrode. Potential control was carried out with a potentiostat (Ivium). Simultaneous absorption spectroscopy was carried out with a halogen white light source (Ocean Optics, DH-2000-BAL) and an optical fiber light path split to separate UV-visible (Ocean Optics, FLAME-S) and near-infrared (Ocean Optics, NQ512) spectrometers. Spectroscopic data were recorded with OceanView software. All analysis was executed with MATLAB software.

### X-ray photoelectron spectroscopy (XPS)

The XPS spectrums of P-x% PTHF were taken using Thermo Scientific ESCALAB 250Xi equipped with a monochromatic KR Al X-ray source (spot size around 500 μm) at the Northwestern University Atomic and Nanoscale Characterization Experimental center (NUANCE). A flood gun was used for charge compensation. The analysis of the spectrum was performed using the Avantage (Thermo Scientific) software.

### Grazing-incidence wide-angle X-ray scattering (GIWAXS)

2D GIWAXS was carried out at the Advanced Photon Source at Argonne National Laboratory on beamline 8-ID-E at room temperature under vacuum with 10.92 keV (*λ* = 1.135 Å) synchrotron radiation with a 0.14° incident angle and measured with a Pilatus 1 M hybrid pixel array detector during 10 s exposures. Data analysis was carried out with GIXSGUI Matlab toolbox^56^.

### Electrical characterization

All the devices are gated by Ag/AgCl electrode through 0.1 M NaCl electrolyte. Basic characterizations like transfer curves and output curves are measured by Keithley 2636B source meter with custom-made LabVIEW programs. For synaptic function characterizations, pulsed gate voltage was generated by Agilent 33210 arbitrary function generator. A Keithley 7001 digital switch was used to change the gate bias between source meter and a function generator. For transient measurement, a DMM 6500 digital multimeter was connected to the source terminal to measure the drain-source current with a sampling rate of 50 kHz.

## Supplementary information

Supplementary Information

## Data Availability

The data that support the findings of this study are available from the corresponding author upon reasonable request.
