# Supplementary material for: Mimicking associative learning using an ion-trapping non-volatile synaptic organic electrochemical transistor
Source: Nat Commun. 2021 Apr 30;12:2480. doi: 10.1038/s41467-021-22680-5 (PMC8087835; doi:10.1038/s41467-021-22680-5)
Supplement: Supplementary file 1 — Supplementary Information [file 41467_2021_22680_MOESM1_ESM.pdf]

Supplementary Information

Mimicking Associative Learning Using an Ion-Trapping Non-Volatile Synaptic Organic Electrochemical Transistor

Xudong Ji[1,2], Bryan D. Paulsen[2], Gary K. K. Chik[1,3], Ruiheng Wu[2], Yuyang Yin[1], Paddy K. L. Chan*[1,3] and Jonathan Rivnay*[2]

Supplementary Figures

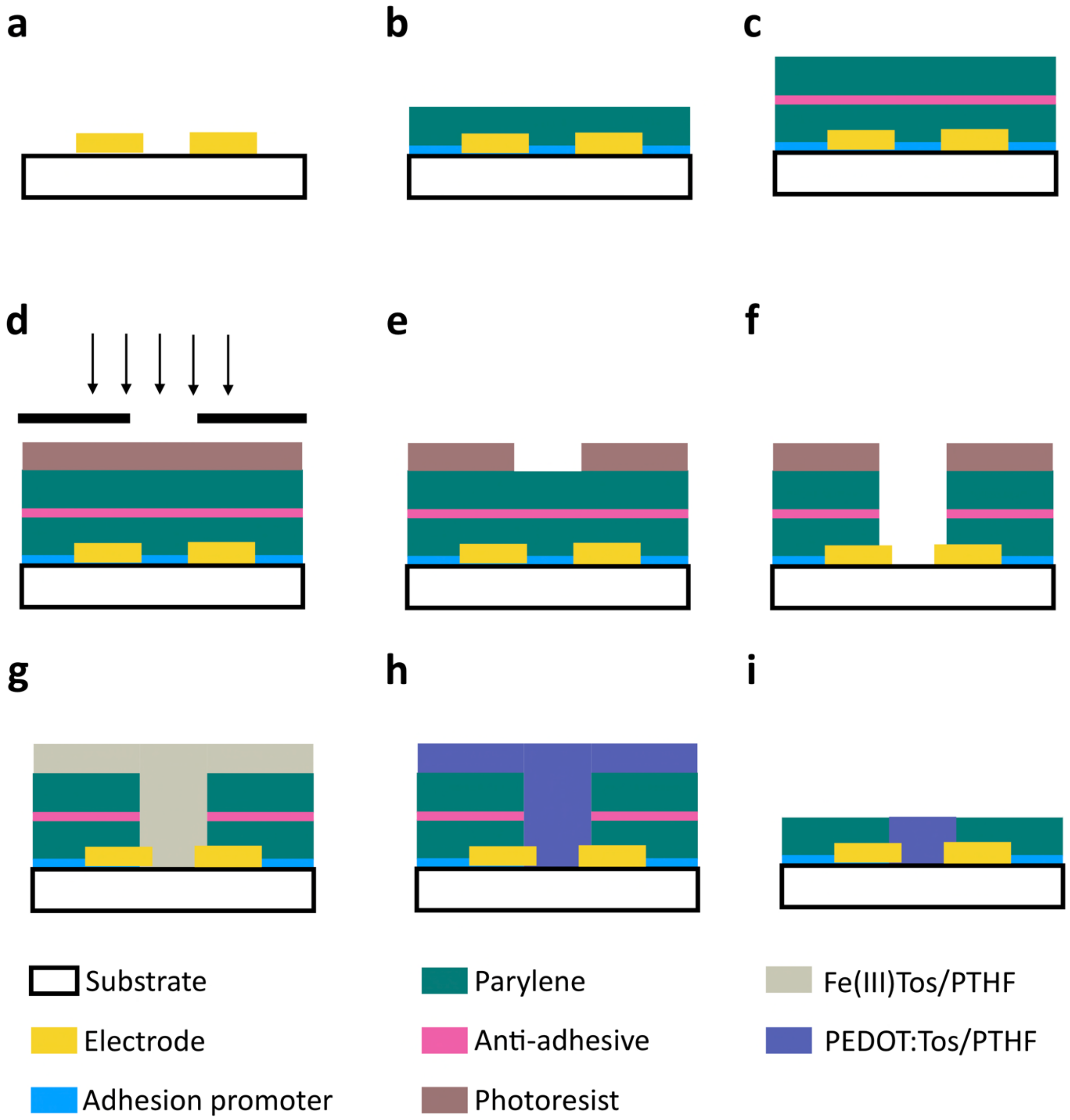

Supplementary Figure 1. The fabrication process of OECT devices.

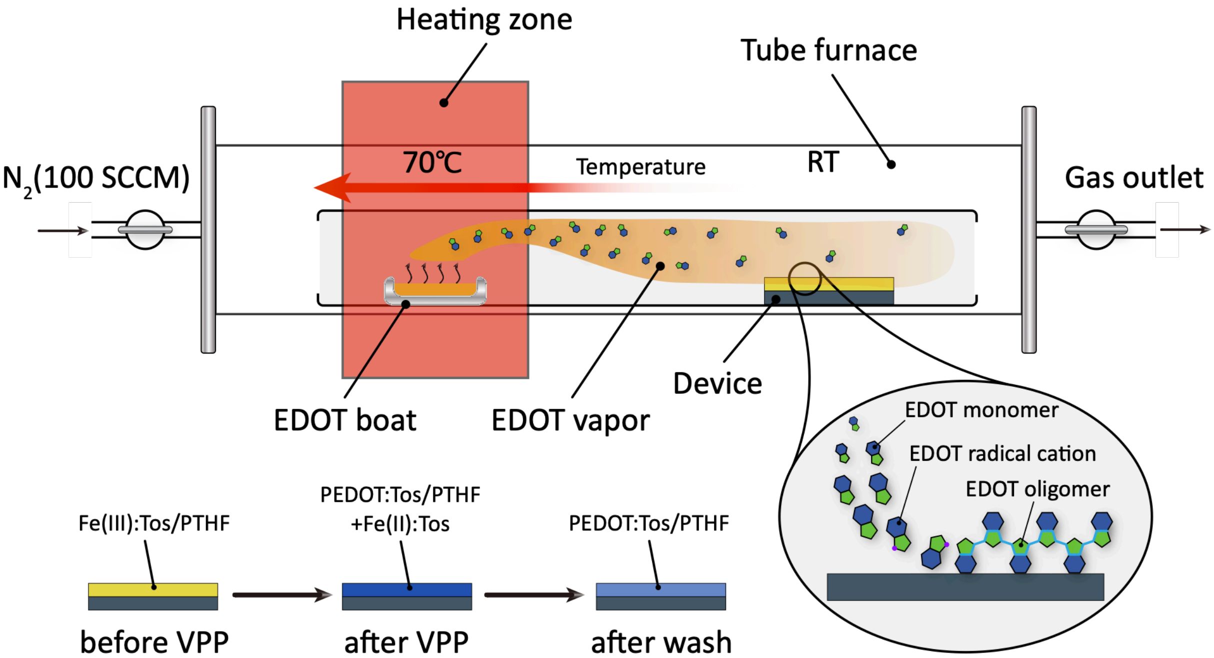

Supplementary Figure 2. VPP process. Schematic illustration of the VPP chamber, the polymerization process of EDOT monomers, and the rinsing of PEDOT:Tos/PTHF film after VPP.

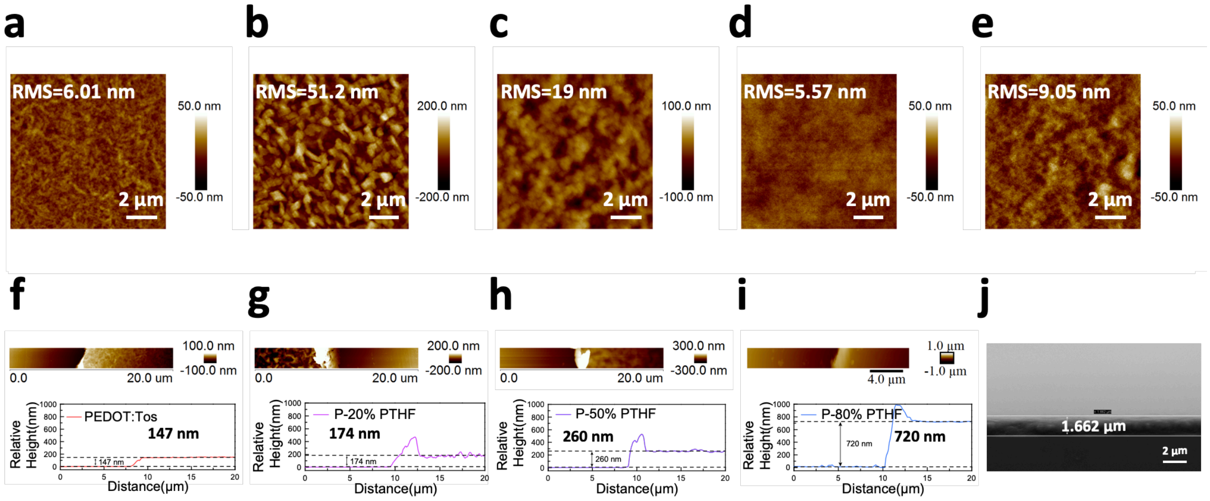

Supplementary Figure 3. Surface morphology and thickness of PEDOT:Tos/PTHF with different compositions. (**a~e**) AFM image of PEDOT:Tos with 0%, 20%, 50%, 80%, 90% PTHF respectively. (**f~i**) Height profile of PEDOT:Tos with 0%, 20%, 50%, 80% PTHF respectively. (**j**) Cross-section SEM image of P-90% PTHF.

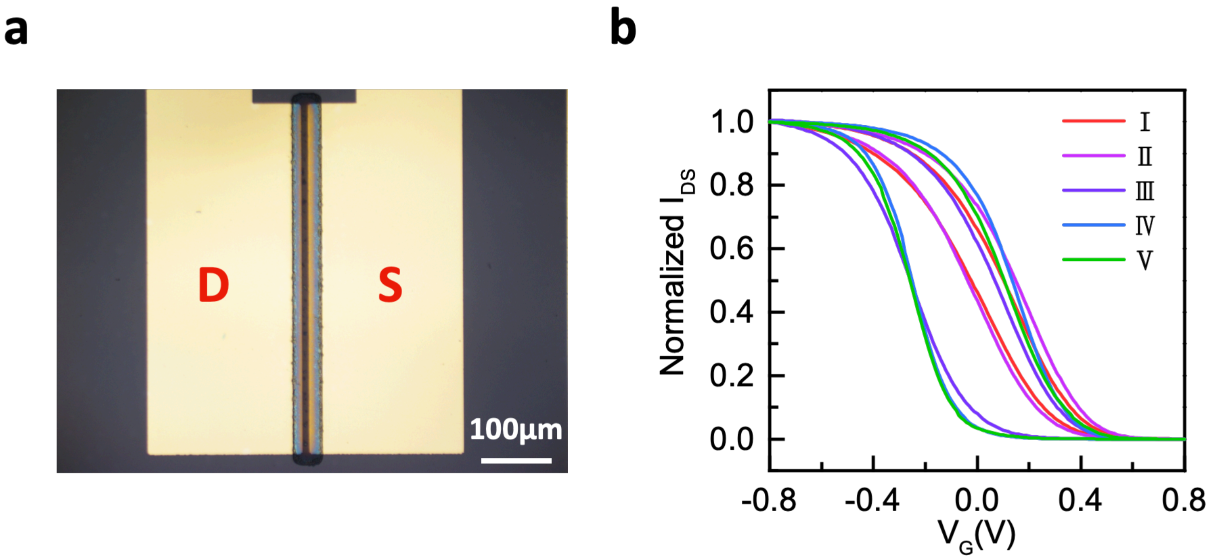

Supplementary Figure 4. Additional characteristics of OECT. (**a**) Optical micrograph of the OECT device. (**b**) Normalized transfer curves of OECT with different channel composition I~V corresponding to PEDOT:Tos loaded with 0%, 20%, 50%, 80%, 90% PTHF respectively.

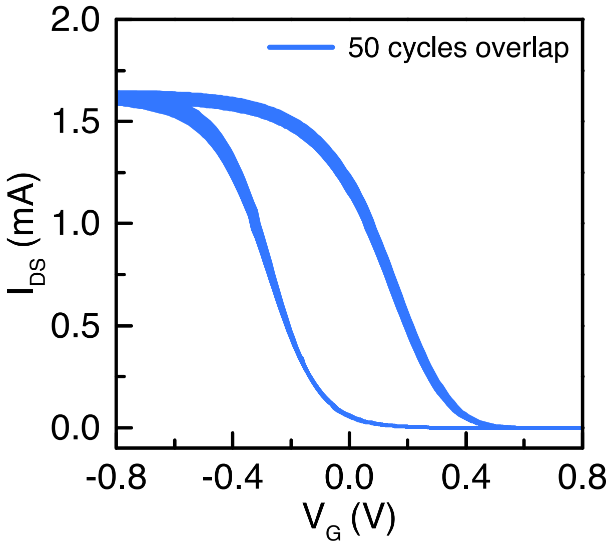

Supplementary Figure 5. Stability. 50 cycles transfer curves of the P-80% PTHF-based OECT overlapped together.

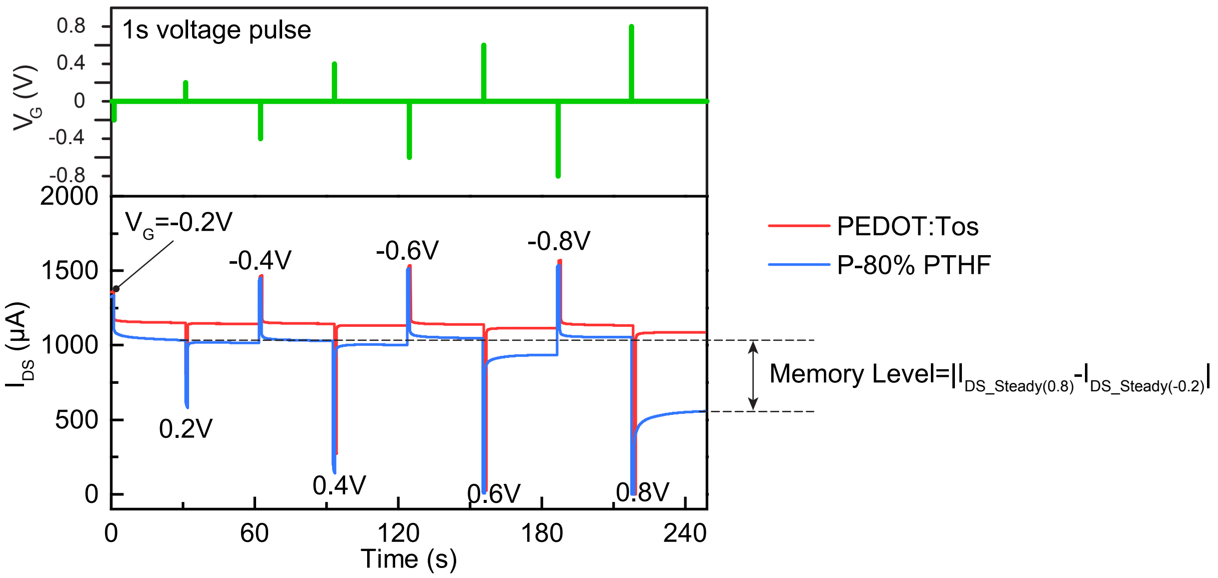

Supplementary Figure 6. Memory level of OECTs. (**a**) Channel current comparison of PEDOT:Tos-based OECT and P-80% PTHF-based OECT after programmed by different gate voltage.

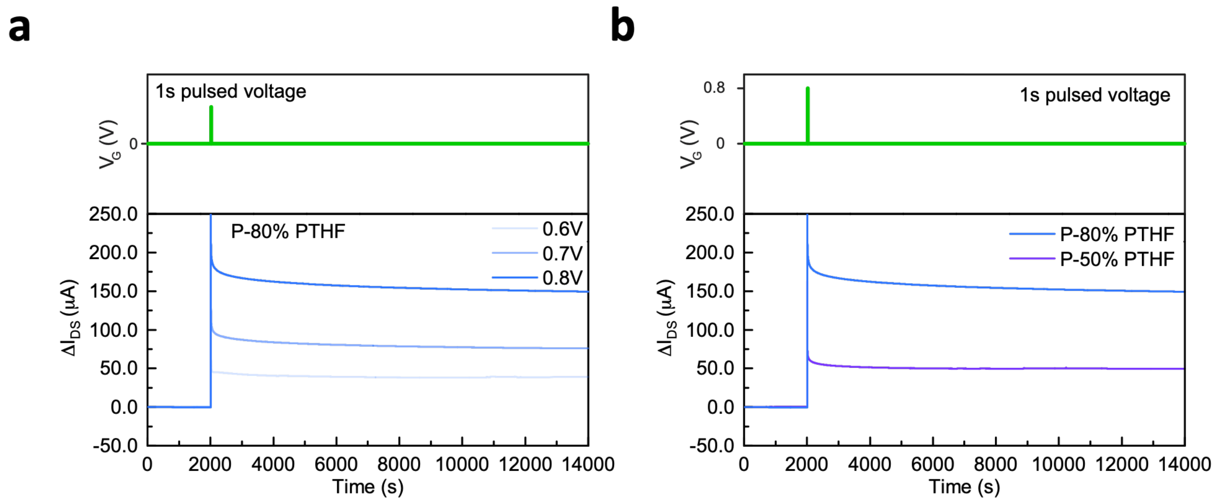

Supplementary Figure 7. Long time memory retention of OECTs. (**a**) Charge retention of P-80% PTHF-based OECT after been biased by 1s pulsed gate voltage with varied amplitude. Retention time is longer than 200 min in all cases. (**b**) Charge retention of both P-50% PTHF and P-80% PTHF-based OECTs after a single gate voltage pulse (1s, 0.8 V). Retention time is longer than 200 min in both devices.

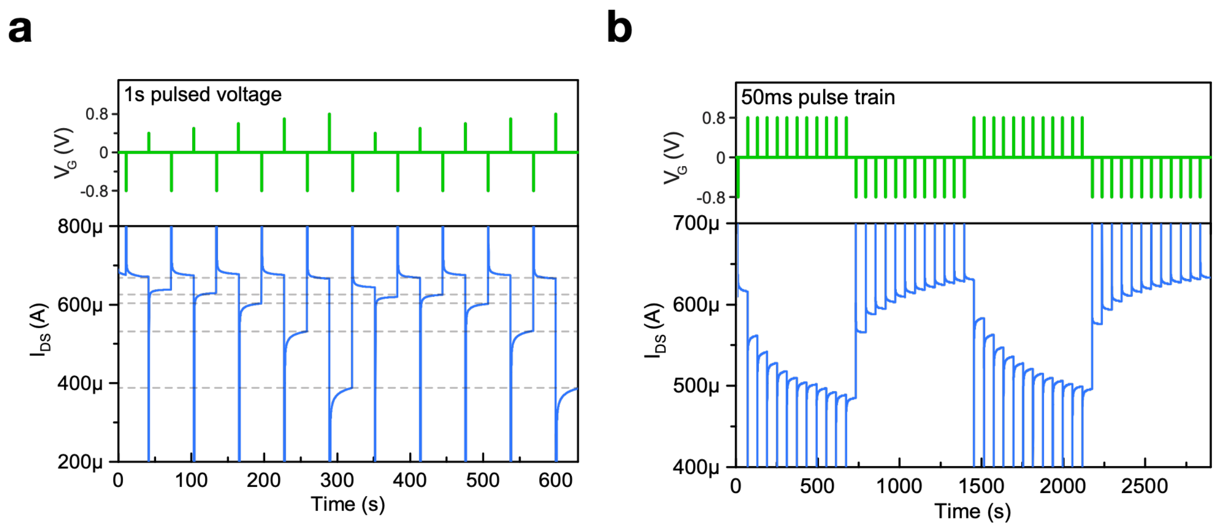

Supplementary Figure 8. Reversibility of OECTs. (**a**) P-80% PTHF-based OECT show discrete channel current value after been biased by pulsed gate voltage with different amplitude and polarity. Highly reproducible channel current can be achieved after the device has been biased by equivalent voltage. (**b**) P-80% PTHF-based OECT shown discrete decreased or increased channel current value in response to a pulse train (V_{DS}=-0.2 V).

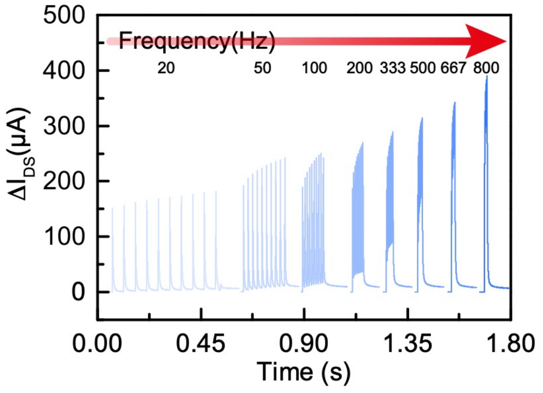

Supplementary Figure 9. Pulsed gate voltage frequency-dependent of channel current modulation in P-80% PTHF-based OECT. The post-tetanic potentiation (PTP) shows a significant increase by increasing the frequency from 20 Hz to 800 Hz.

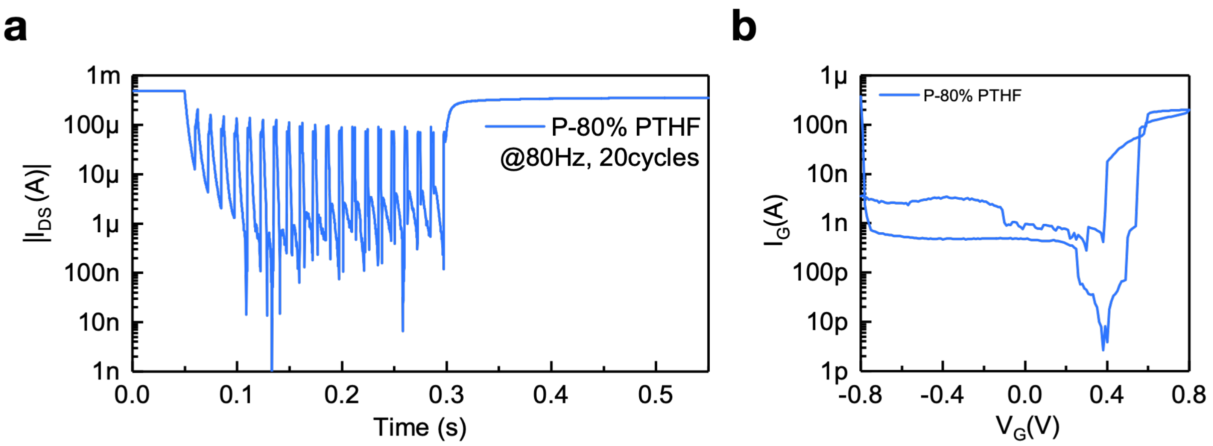

Supplementary Figure 10. I_{DS} in log-scale and leakage current. (**a**) Channel current as a function of time under 20 cycles pulsed gate voltage in a log-scale. (**b**) Gate leakage current of P-80% PTHF-based OECT from a transfer curve sweep.

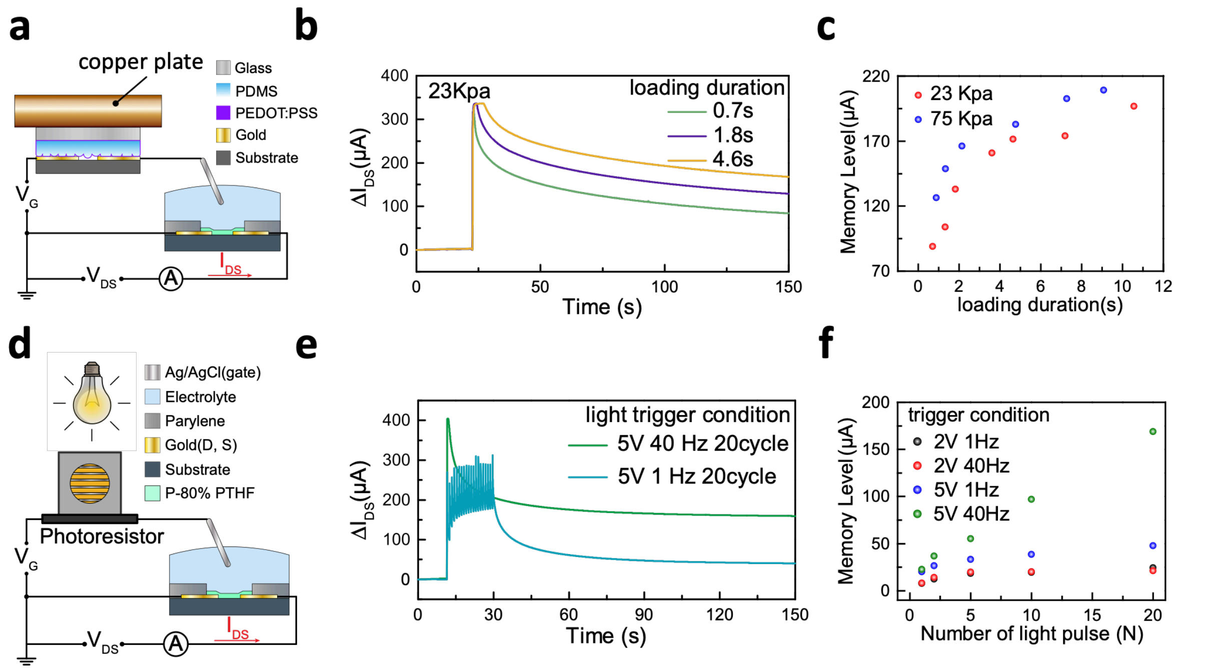

Supplementary Figure 11. Haptic and iconic memory in OECT. (**a**) Schematic illustration of the integration of the pressure sensor with the OECT device. A copper plate was used as the external pressure stimulus. (**b**) Channel current change triggered by the same pressure with different duration. (**c**) Memory level of the device as a function of pressure amplitude and duration. (**d**) Schematic illustration of the integration of the photoresistor with the OECT device. The LED bulb was used as the external light stimulus. (**e**) Channel current change triggered by light with different frequencies. (**f**) Memory level of the device as a function of light intensity, frequency, and the number of light pulses.

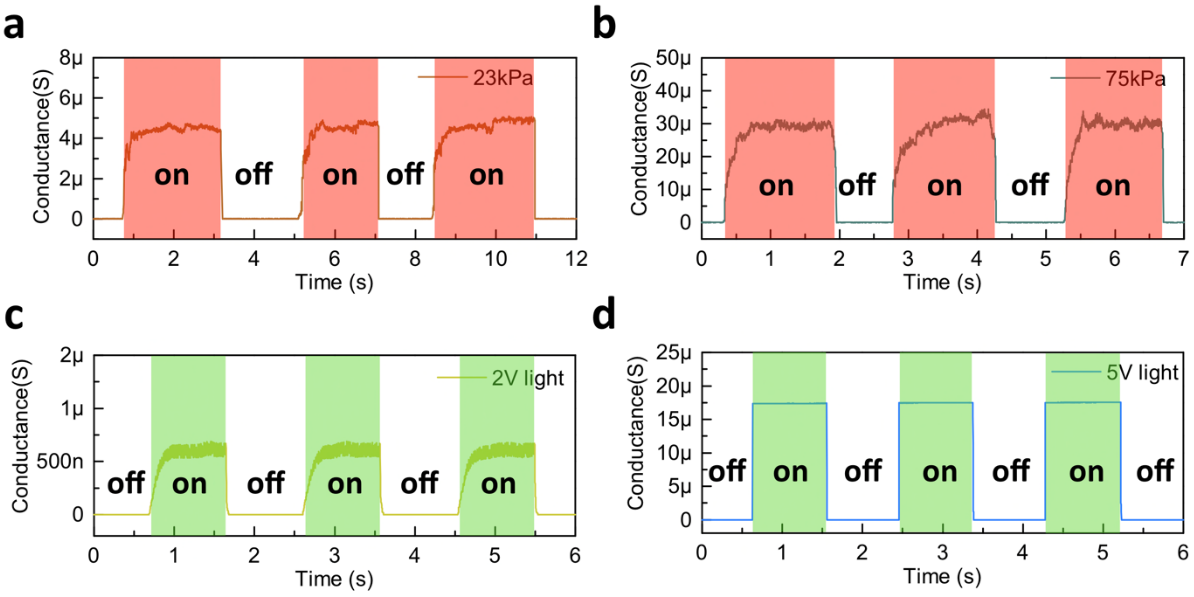

Supplementary Figure 12. Characterization of pressure sensor and photoresistor (**a**, **b**) Conductance of pressure sensor after triggered by 23 kPa and 75 kPa pressure respectively. (**c**, **d**) Conductance of photoresistor after triggered by LED biased by 2 V and 5 V voltage respectively

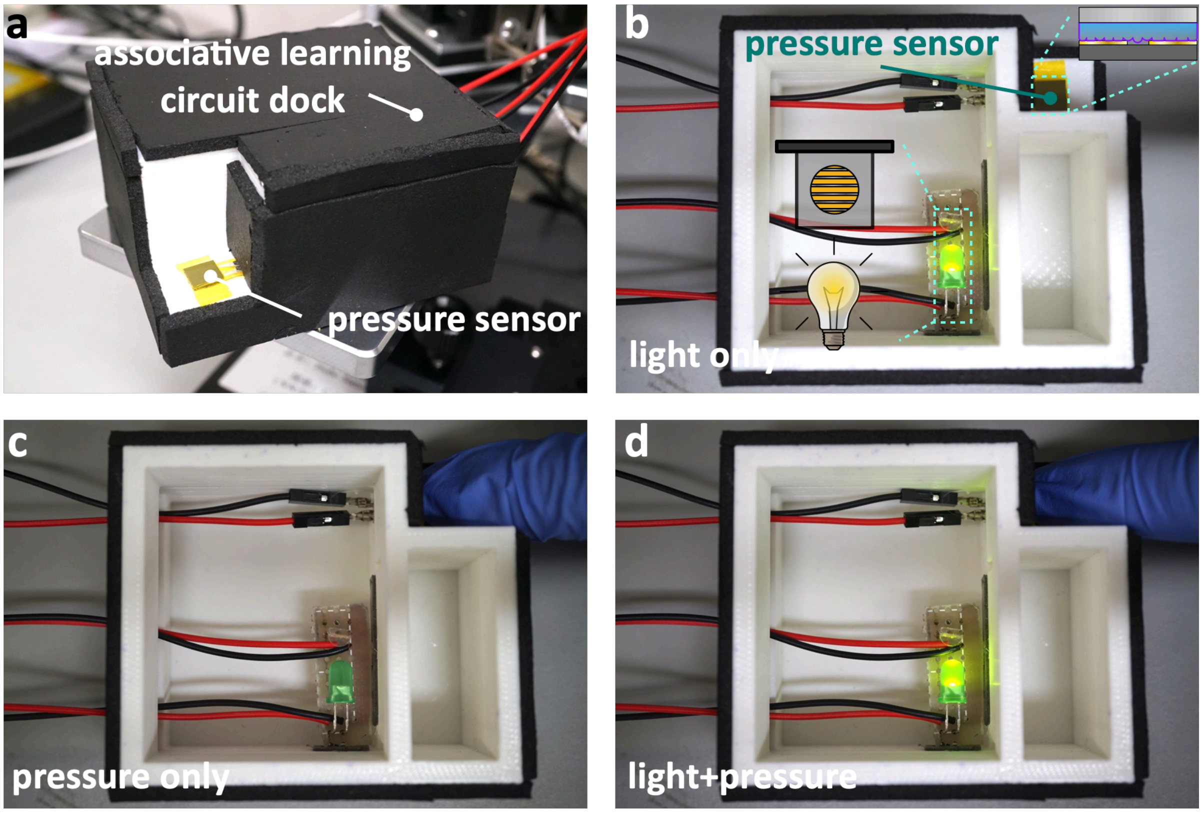

Supplementary Figure 13. Associative learning dock. (**a**) Photograph of associative learning dock. (**b~d**) Different stimuli conditions on the neuromorphic circuit. (**b**) light only (CS); (**c**) pressure only (US): (**d**) light and pressure together (training process).

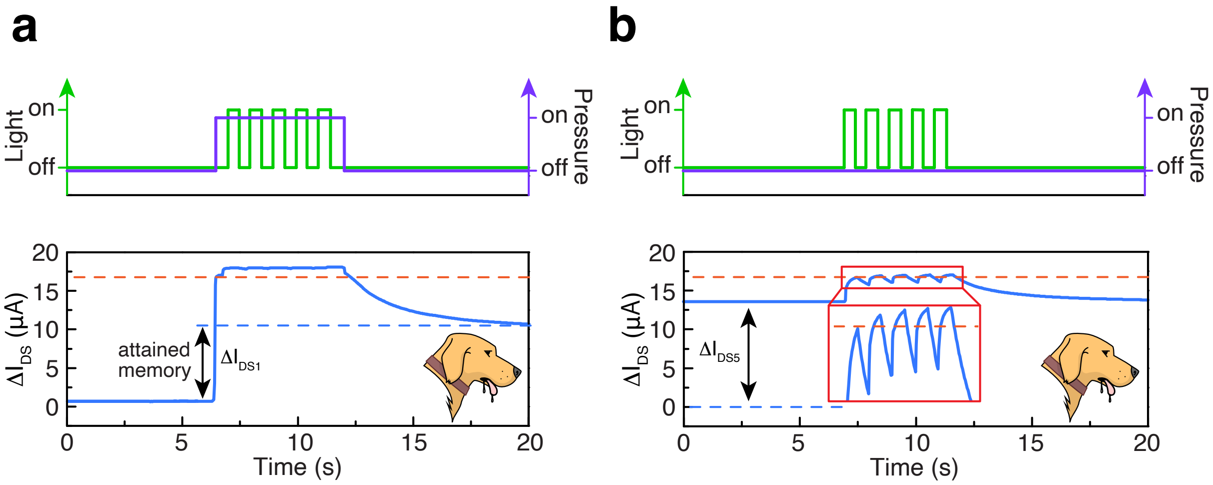

Supplementary Figure 14. Enlarged details in associative learning process. (**a**) The first training of the neuromorphic circuit by applying the US and the CS simultaneously; the channel current change reached the memory threshold, triggering the UR, and was partially retained (ΔI_{DS1}) after removing the CS and US. (**b**) Channel current change caused by the CS after the fifth training; the amplitude reached the memory threshold (shown in insert) and successfully caused the UR. ΔI_{DS5} indicate the attained memory induced by the five consecutive training processes.

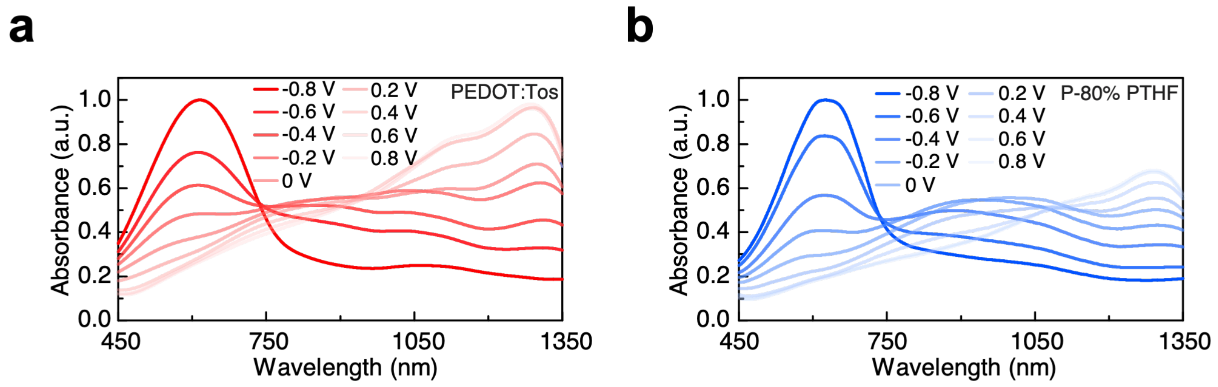

Supplementary Figure 15. Additional UV-Vis-NIR results. (**a**, **b**) UV-Vis-NIR absorbance of PEDOT:Tos and P-80% PTHF during electrical bias.

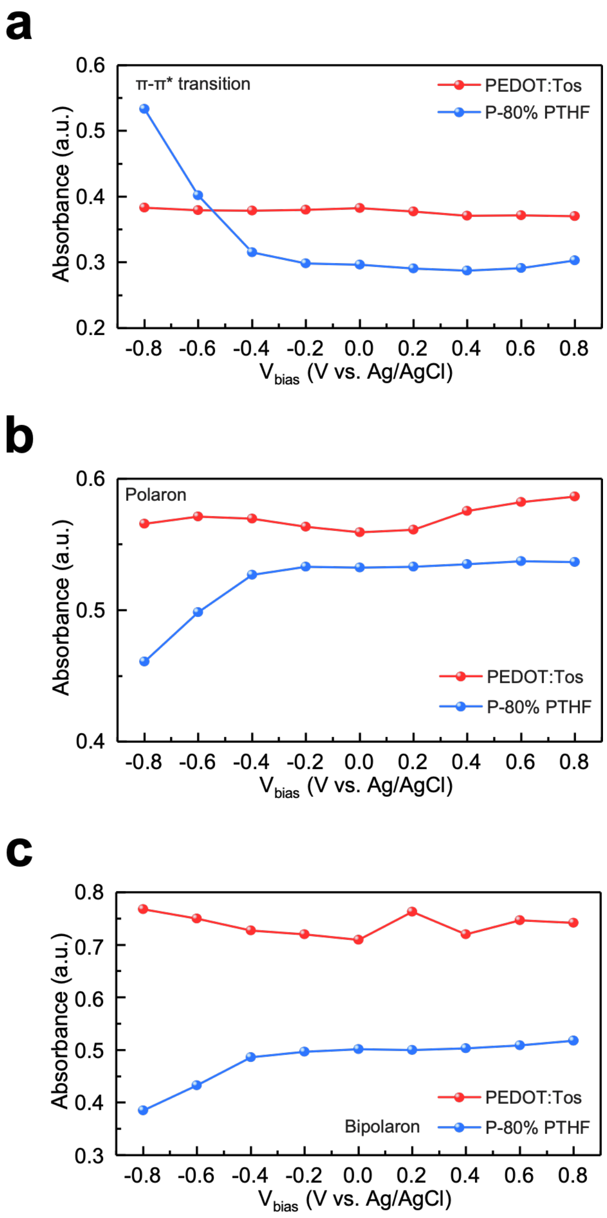

Supplementary Figure 16. Quantitative comparison of absorbance between PEDOT:Tos and P-80% PTHF. (**a**) Comparison of π-π* transition absorbance between PEDOT:Tos and P-80% PTHF with respect to the program bias. (**b**) Comparison of polaron absorbance between PEDOT:Tos and P-80% PTHF with respect to the program bias. (**c**) Comparison of bipolaron absorbance between PEDOT:Tos and P-80% PTHF with respect to the program bias.

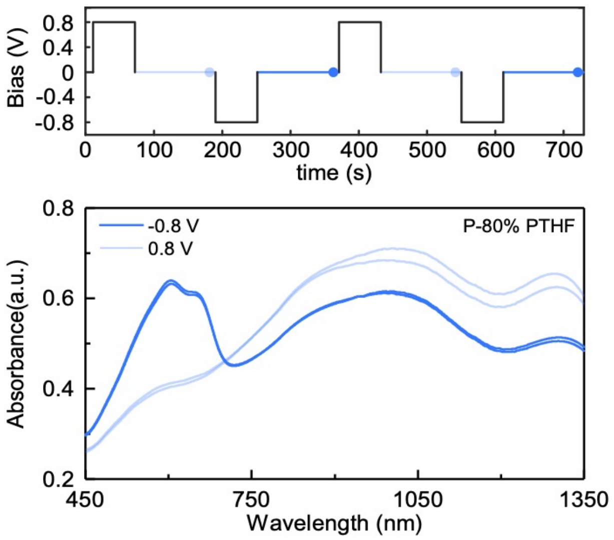

Supplementary Figure 17. Reversibility of the non-volatile oxidation level change in P-80% PTHF induced by electrical bias.

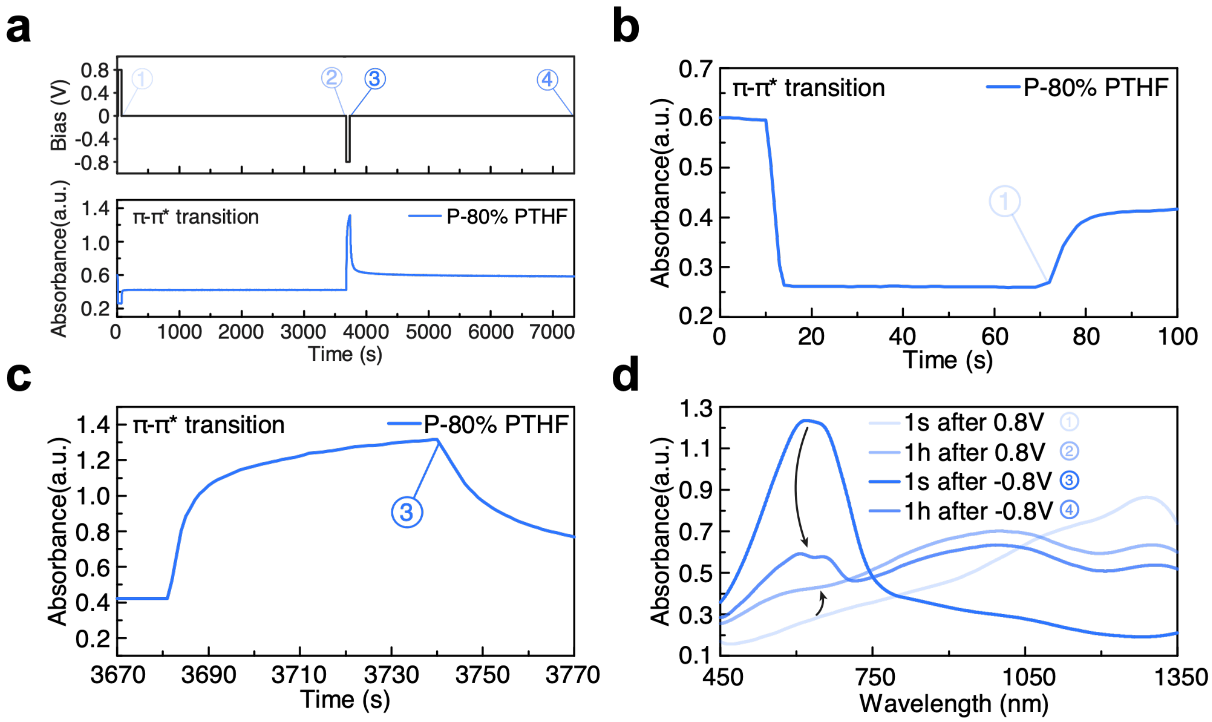

Supplementary Figure 18. Time-resolved π-π* transition change of P-80% PTHF. (**a**) intensity of π-π* transition with respect to time under defined bias pattern. (**b**, **c**) enlarged view of π-π* transition. (**d**) long-term stability of the non-volatile nature of P-80% PTHF under low and high oxidation level in electrolyte.

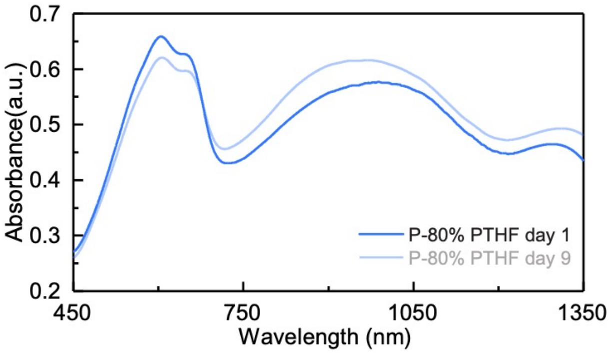

Supplementary Figure 19. Long-term stability. Absorbance comparison of P-80% PTHF being biased by -0.8 V for 10s in day 1 and day 9.

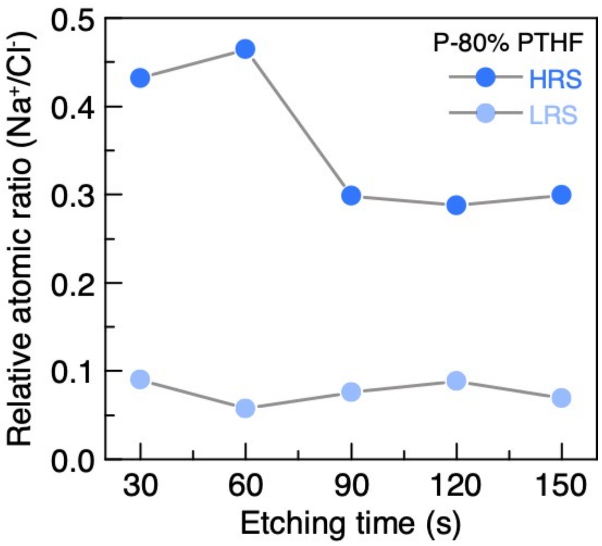

Supplementary Figure 20. Depth profile of P-80% PTHF tracking the relative ratio of Na^{+} over Cl^{-}.

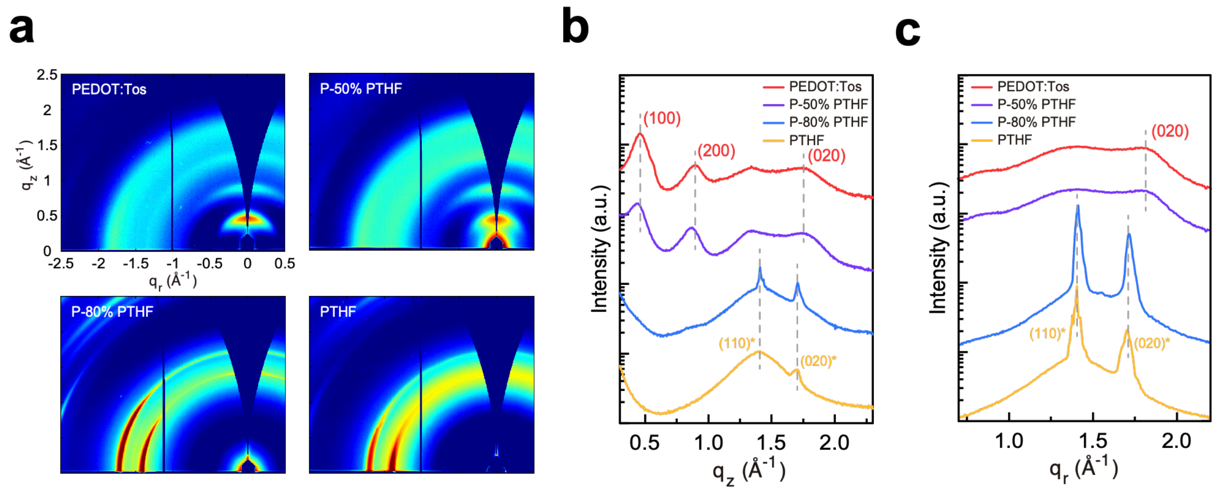

Supplementary Figure 21. GIWAXS of as-cast P-x% PTHF films. (**a**) GIWAXS patterns of PEDOT:Tos/PTHF films with different composition. (**b**, **c**) Out-of-plane (q_z) and in-plane (q_r) linecuts of the PEDOT:Tos/PTHF films with different composition.

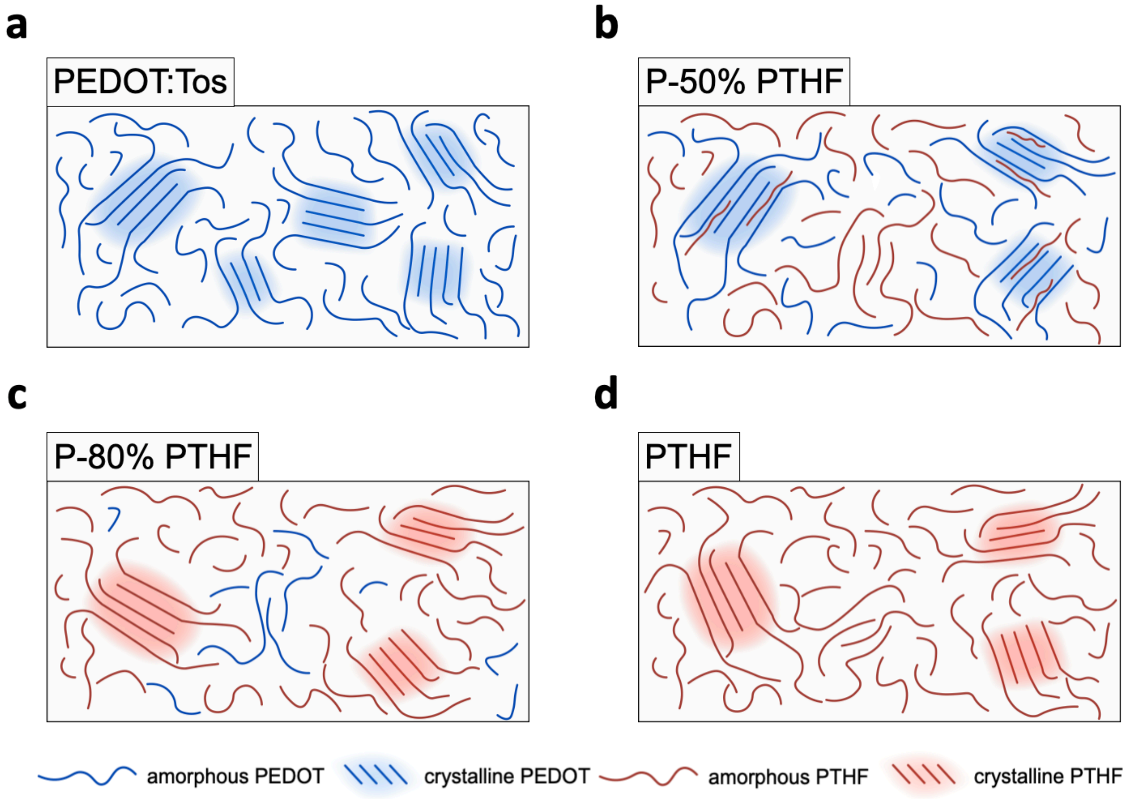

Supplementary Figure 22. Schematic image of the microstructure of PEDOT:Tos/PTHF composite with different composition.

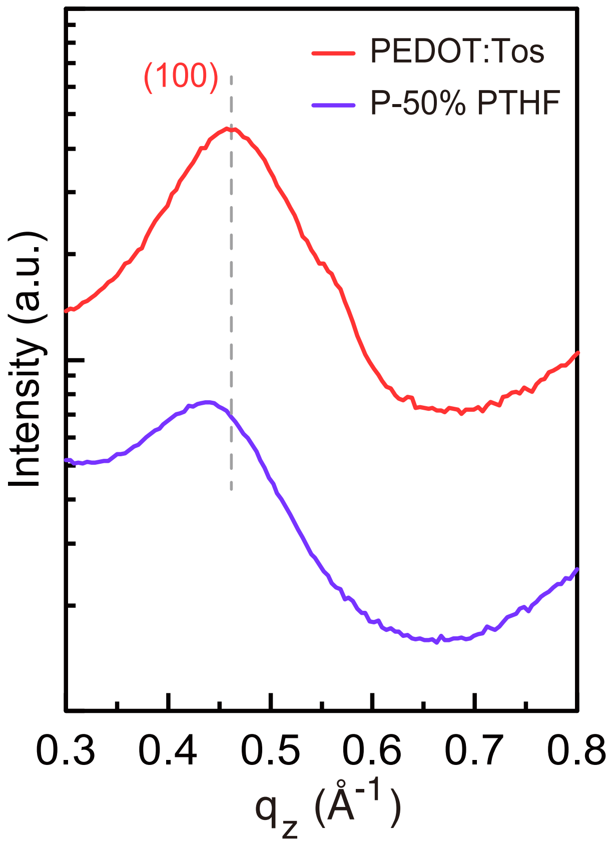

Supplementary Figure 23. Enlarged out-of-plane linecuts of PEDOT:Tos and P-50% PTHF. A negative shift of diffraction peak in P-50% PTHF can be observed.

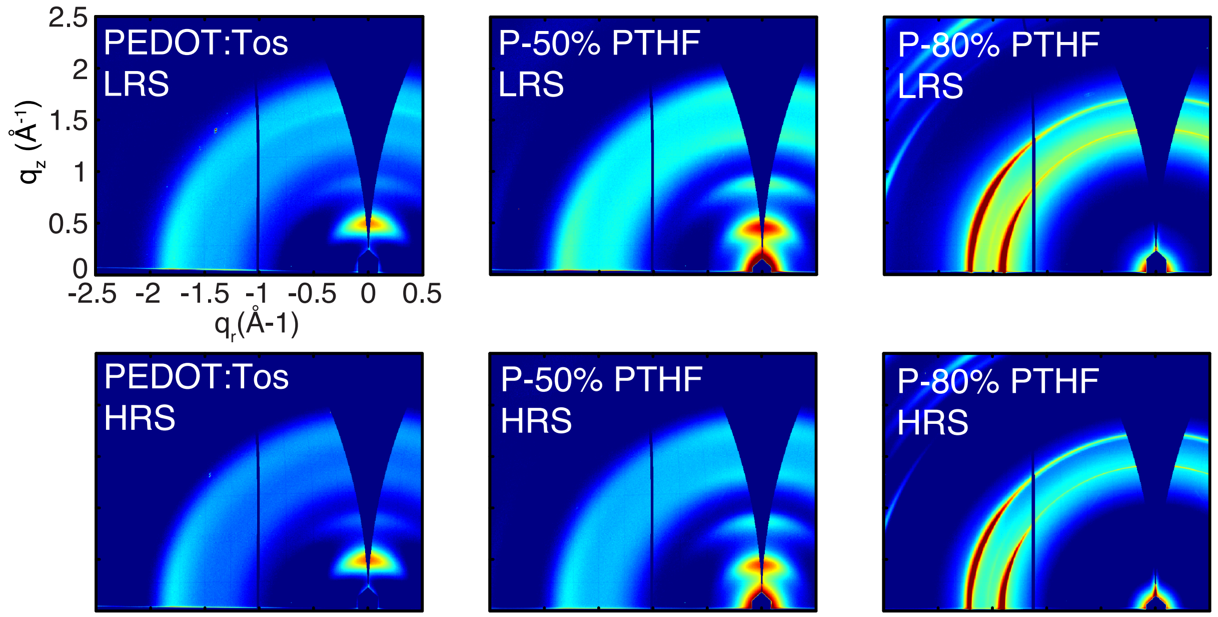

Supplementary Figure 24. GIWAXS patterns of PEDOT:Tos, P-50% PTHF and P-80% PTHF films in LRS and HRS.

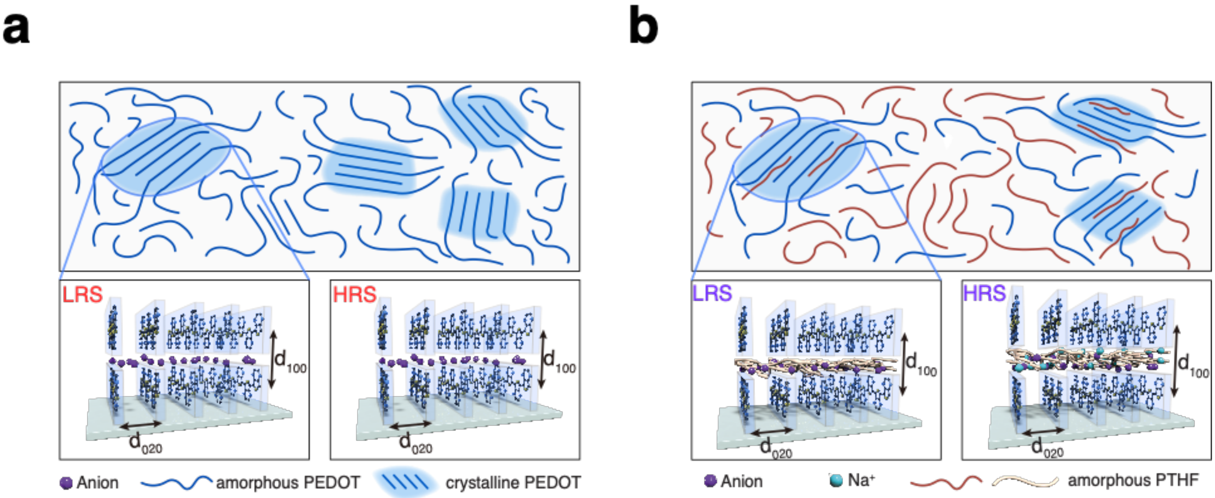

Supplementary Figure 25. Microstructure of PEDOT:Tos and P-50% PTHF. (**a**, **b**) Schematic images of microstructure change in PEDOT:Tos and P-50% PTHF under LRS and HRS; d_{100} indicate the lamellar staking distance and d_{020} indicate the π-π stacking distance. Lamella spacing expanded for P-50% PTHF in HRS compared with LRS owing to the trapped Na^{+}.

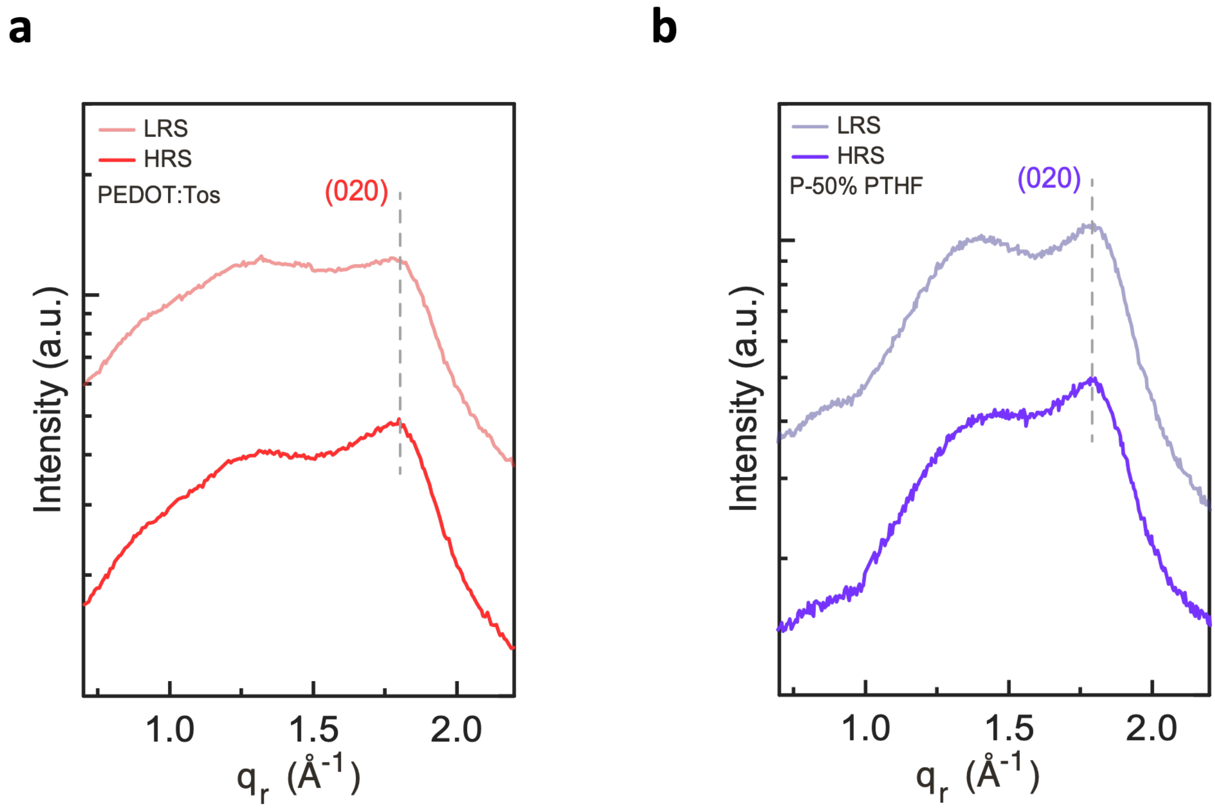

Supplementary Figure 26. In-plane linecuts of PEDOT:Tos and P-50% PTHF films in LRS and HRS.

Supplementary Tables

Supplementary Table 1. Fitting parameters for PPF index.

PTHF percentage	C_1(%)	τ_1 (ms)	C_2(%)	τ_2 (ms)
0	\	\	\	\
20%	1.48	0.55	0.61	5.82
50%	9.26	1.50	0.99	64.80
80%	34.98	2.22	8.37	124.74
90%	34.41	7.16	21.97	57.14

Supplementary Table 2. Fitting parameters for PTP index.

PTHF percentage	C_1(%)	τ_1 (ms)	C_2(%)	τ_2 (ms)
0	\	\	\	\
20%	2.30	0.21	1.81	5.08
50%	15.2	0.96	5.18	18.73
80%	60.99	2.32	31.31	121.91
90%	279.6	3.36	119.5	31.25

Supplementary Table 3. Mass and volume of different components in PEDOT:Tos/PTHF precursor.

PTHF percentage	PTHF (g)	Fe(III):Tos (g)	Butanol (g)	Pyridine (μL)
0	0	1	4	60
20%	0.023	1	3.977	60
50%	0.093	1	3.907	60
80%	0.37	1	3.63	60
90%	0.84	1	3.16	60

Supplementary Note

Supplementary Note 1. Artificial perception for haptic and iconic memory

Haptic memory and iconic memory are two basic types of human sensory memory that originate from tactile and visual stimuli. Through these two types of memory, individuals can retain the impression of these sensations even after the termination of the external stimuli.[1,2] A pressure sensor or a photoresistor was used to transduce the tactile or visual stimuli into electrical signals, respectively. Then, these electrical signals were further processed by our non-volatile synapse-like OECT devices. Firstly, a tactile perception system for simulating haptic memory was achieved by integrating a micro-hump PDMS-based resistive pressure sensor we previously developed[3] on the gate terminal of the OECT with a 0.7 V constant bias as shown in Supplementary Fig. 11a. The varying resistance state of the pressure sensors resulted in different effective gate voltage applied on the active channel of the OECT. Because of the non-volatile memory property of our P-80% PTHF-based OECT, after pressure was removed, a specific channel current difference can be "memorized" by the OECT device depending on the amplitude and duration of the applied pressure. Two copper plates with different weights (23 kPa and 75 kPa) were placed on the pressure sensor to mimic the pressure loading. As shown in Supplementary Fig. 12a and b, the conductance of the pressure sensor is 4.5 μS and 30 μS under various 23 kPa and 75 kPa pressures, respectively. ΔI_{DS} triggered by the pressure of varying duration is shown in Supplementary Fig. 11b, and the results indicate a pressure duration-dependent property. The detailed relationship of pressure-induced haptic memory with the duration and amplitude of pressure is summarized in Supplementary Fig. 11c. The memory level is defined as the ΔI_{DS} value after the copper plates were removed for 2 minutes. It can be seen that the haptic memory level elevated when higher pressures with longer duration were applied. In a similar way to our simulation of haptic memory, visual stimuli-induced iconic memory was simulated by integrating a commercially available photoresistor

instead of the pressure sensor as shown in Supplementary Fig. 11d. A LED bulb was put aside the photoresistor as a light source whose intensity can be adjusted by different voltages. As can be seen from Supplementary Fig. 12c and d, the conductance of the photoresistor was around 0.6 μS and 17.5 μS when the LED bulb was biased at 2 V and 5 V, respectively. The LED bulb can be triggered by a highly controllable voltage pattern through the function generator to achieve a programmable light pattern with various light intensity, frequency, and cycles. These different light patterns can be used as diverse visual stimuli, which helped us to study the relationship between different visual stimuli and their corresponding iconic memory. Two sets of voltage pulses with different frequencies (1 Hz and 40 Hz) but with the same duration (20 ms), amplitude (5 V) and cycle number (N=20) were applied on the LED bulb; the resulting channel current change can be observed in Supplementary Fig. 11e, which shows that visual stimuli with higher frequency can trigger an increase in iconic memory. All three parameters of light generated from the LED bulb including intensity, frequency, and cycles could affect the resulted iconic memory level, which is summarized in Supplementary Fig. 11f. It is evident that a higher iconic memory level can be obtained by triggering the system with light possessing a greater intensity, a higher frequency as well as more cycles.

Supplementary References

1 Gordon, A. M., Westling, G., Cole, K. J. & Johansson, R. S. Memory representations underlying motor commands used during manipulation of common and novel objects. *J. Neurophysiol.* **69**, 1789-1796 (1993).

2 Hillis, J. M., Ernst, M. O., Banks, M. S. & Landy, M. S. Combining sensory information:mandatory fusion within, but not between, senses. *Science* **298**, 1627-1630 (2002).

3 Wang, Z. *et al.* High Sensitivity, Wearable, Piezoresistive Pressure Sensors Based on Irregular Microhump Structures and Its Applications in Body Motion Sensing. *Small* **12**, 3827-3836 (2016).
